# Chiral Polymers Based on Vinyl[2.2]paracyclophane and Their Application as CPL Emitters

**DOI:** 10.3390/polym17081070

**Published:** 2025-04-16

**Authors:** Henrik Tappert, Emma V. Puttock, Jhon Sebastian Oviedo Ortiz, Eli Zysman-Colman, Jeanne Crassous, Stefan Bräse

**Affiliations:** 1 Institute of Organic Chemistry (IOC), Karlsruhe Institute of Technology (KIT), Kaiserstraße 12, 76131 Karlsruhe, Germanyemma.puttock@kit.edu (E.V.P.); 2 Institute of Biological and Chemical Systems-Functional Molecular Systems (IBCS-FMS), Karlsruhe Institute of Technology (KIT), Kaiserstraße 12, 76131 Karlsruhe, Germany; 3 Institut des Sciences Chimiques de Rennes, Univ Rennes, Unité Mixte de Recherche (UMR) Centre National de la Recherche Scientifique (CNRS) 6226, Campus de Beaulieu, 35042 Rennes, CEDEX, Francejeanne.crassous@univ-rennes.fr (J.C.); 4 Organic Semiconductor Centre, EaStCHEM School of Chemistry, University of St Andrews, Fife, St Andrews KY16 9ST, UK; eli.zysman-colman@st-andrews.ac.uk

**Keywords:** chiral polymer, [2.2]paracyclophane, polyvinyl, CPL

## Abstract

Chiral molecules are integral to various biological and artificial systems, influencing processes from chemical production to optical activities. In this study, we explore the potential of chiral vinyl[2.2]paracyclophane (vinyl-PCP) as a monomer for the synthesis of homopolymers and copolymers with styrene. We achieved polymerization through anionic, cationic, and radical methods. The resulting polymers demonstrated significant chiral properties, even in copolymers with small fractions of the chiral monomer. Further, we developed a polymerizable vinyl emitter from 10-(4-(4,6-diphenyl-1,3,5-triazin-2-yl)phenyl)-9,9-dimethyl-9,10-dihydroacridine (DMAC-TRZ) through a two-step synthesis with an overall yield of 48%. Copolymerization with chiral vinyl-PCP resulted in emissive polymers that demonstrated circularly polarized luminescence (CPL) properties. The inclusion of the chiral PCP monomer, acting both as a host material and the source of chirality for CPL, enhanced the photoluminescence quantum yield (PLQY) to 47.2% in N_2_ at 5–10% emitter content, compared to 26.8% for the pure emitter polymer. CPL-active polymers show clear mirror-image Cotton effects at 240 nm and 267 nm and dissymmetry factors around +2 × 10^−4^ and −1 × 10^−4^. This self-hosting effect of PCP monomers underscores the potential of chiral vinyl-PCP for advanced functional materials in optical communication and bio-responsive imaging.

## 1. Introduction

Chiral molecules play key roles in many areas of human life, whether in biological systems such as the human body or in artificial systems like the chemical production of a wide range of molecules [1,2,3,4,5]. Even light output is influenced by these “optically active” molecules and can be polarized, both circularly and linearly, to interact selectively with chiral matter [6]. Consequently, new molecules that can influence and control the chirality in the above-mentioned fields are highly sought after. Functional materials made from chiral polymers can help with separating [7], catalyzing [8], and sensing specific enantiomers [9] or may even help in up-and-coming fields such as optical communication [10] or bio-responsive imaging [11]. One of the possibilities to achieve polymers with chiral properties is using chiral monomers, transferring their chirality to the macromolecule [12]. One possible monomer for this is a functionalized PCP.

PCPs exhibit planar chirality upon substitution, and small molecules using this building block have been successful in multiple fields [13], many of them requiring chirality, such as asymmetric catalysis [14], medical science [15], and chiroptical emitters [16,17,18]. While PCP polymers are known in the literature, the term often refers to parylenes that result from breaking PCP, and with them, their chirality [19]. In many of the cases where the PCP was kept intact, achiral substitution patterns or racemic mixtures were used [20,21]. For this reason, chiral PCP polymers suitable for the above applications are rare. In this study, we explore a possible building block for chiral polymers in these applications: vinyl[2.2]paracyclophane. The vinyl group is a long-known moiety that can be used in various polymerizations. Although the use of vinyl-PCP for polymers has been reported in a single study, it was only used as a racemic mixture and has not been employed in any application [22]. In this work, we aimed to utilize the enantiomerically pure synthesis of vinyl-PCP previously reported by our group [23] to form the corresponding chiral polymer and progress to the next step: exploring the behavior of the chirality in copolymerization with styrene as a model system, as well as with a new vinyl-substituted variant of the emitter DMAC-TRZ [24] to show its application as a functional material.

## 2. Materials and Methods

### 2.1. General Remarks

The starting materials, solvents, and reagents were purchased at 97–99% purity from abcr (Karlsruhe, Germany), Acros (Geel, Belgium), Bernd Kraft (Duisburg, Germany), arbosynth (Redcar, United Kingdom), ChemPUR (Karlsruhe, Germany), Honeywell (Offenbach, Germany), Merck (Darmstadt, Germany), Sigma Aldrich (St. Louis, MO, USA), TCI (Tokyo, Japan), and Thermo Fisher Scientific (Waltham, MA, USA) and used without further purification unless stated otherwise.

Solvents of technical quality were purified by distillation or with an MB SPS5 solvent purification system (acetonitrile, dichloromethane (DCM), diethyl ether, tetrahydrofuran (THF), toluene) from MBraun (Garching, Germany). Solvents of p.a. quality were purchased from Acros (Geel, Belgium), Fisher Scientific (Waltham, MA, USA), Sigma Aldrich (St. Louis, MO, USA), Roth (Karlsruhe, Germany), or Riedel-de Haën (Seelze, Germany) and were used without further purification. Spectroscopic grade toluene was purchased from VWR (Darmstadt, Germany) and used without further purification.

Air- and moisture-sensitive reactions were carried out under an argon atmosphere in oven-dried glassware using standard Schlenk techniques.

Liquids were added with a stainless-steel cannula, and solids were added in powdered shape.

Reactions at low temperatures were cooled using flat dewars produced by Isotherm (Karlsruhe, Germany) with water/ice or isopropanol/dry ice mixtures.

Solvents were evaporated under reduced pressure at 45 °C using a rotary evaporator. For solvent mixtures, each solvent was measured volumetrically.

Flash column chromatography was performed using Merck (Darmstadt, Germany) silica 60 (0.040 × 0.063 mm, 230–400 mesh ASTM) and quartz sand (glowed and purified with hydrochloric acid).

### 2.2. Reaction Monitoring

All reactions were monitored by thin-layer chromatography using silica-coated aluminum plates (Merck (Darmstadt, Germany), silica 60, F254). UV-active compounds were detected with a UV lamp at 254 nm and 366 nm excitation.

### 2.3. Melting Point

Melting points were detected on an OptiMelt MPA100 device from the Stanford Research System (Sunnyvale, CA, USA).

### 2.4. Optical Rotation

Optical rotation was measured with a Perkin Elmer (Rodgau, Germany) 241 Polarimeter using a 100 mm glass cell, a suitable solvent at the sodium D lines (589.0 and 589.6 nm), and a constant temperature of 20 °C.

### 2.5. Nuclear Magnetic Resonance Spectroscopy (NMR)

NMR spectra were recorded on a Bruker (Karlsruhe, Germany) Avance 400 NMR instrument at 400 MHz for ^1^H NMR and 101 MHz for ^13^C NMR.

The NMR spectra were recorded at room temperature in deuterated solvents acquired from Eurisotop (Saint-Aubin, France). The chemical shift *δ* is displayed in parts per million [ppm], and the references we used were the ^1^H and ^13^C peaks of the solvents themselves as follows:

*d*_1_-chloroform (CDCl_3_): 7.26 ppm for ^1^H and 77.16 ppm for ^13^C

*d*_2_-dichloromethane (CD_2_Cl_2_): 5.32 ppm for ^1^H and 53.84 ppm for ^13^C

For the characterization of centrosymmetric signals, the signal’s median point was chosen for multiplets in the signal range. The following abbreviations were used to describe the proton splitting pattern: d = doublet, t = triplet, m = multiplet, dd = doublet of a doublet, ddd = doublet of a doublet of a doublet, dddd = doublet of a doublet of a doublet of a doublet, and dt = doublet of a triplet. Absolute values of the coupling constants “*J*” are given in Hertz [Hz] in absolute value and decreasing order. Signals of the ^13^C spectrum were assigned by distortionless enhancement by polarization transfer (DEPT) spectra DEPT90 and DEPT135 or phase-edited heteronuclear single quantum coherence (HSQC). They were specified in the following way: + = primary or tertiary carbon atoms (positive phase), − = secondary carbon atoms (negative phase), and C_q_ = quaternary carbon atoms (no signal).

### 2.6. Infrared Spectroscopy (IR)

The infrared spectra were recorded with a Bruker (Karlsruhe, Germany) Alpha P instrument. All samples were measured by attenuated total reflection (ATR). The positions of the absorption bands are given in wavenumbers $\tilde{v}$ in cm^−1^ and were measured in the range from 3600 cm^−1^ to 500 cm^−1^.

Characterization of the absorption bands was performed in dependence of the absorption strength with the following abbreviations: vs (very strong, 0–9%), s (strong, 10–39%), m (medium, 40–69%), w (weak, 70–89%), and vw (very weak, 90–100%).

### 2.7. Mass Spectrometry (MS)

Electron ionization (EI) and fast atom bombardment (FAB) experiments were conducted using a Finnigan MAT (Bremen, Germany) 95 (70 eV) instrument, with 3-nitrobenzyl alcohol (3-NBA) as the matrix and reference for high resolution. For the interpretation of the spectra, molecular peaks [M]^+^, the peaks of protonated molecules [M + H]^+^, and characteristic fragment peaks are indicated with their mass-to-charge ratio (*m*/*z*), and their intensity in percent, relative to the base peak (100%), is given.

### 2.8. Gel Permeation Chromatography (GPC)

For GPC measurements, a PSS SECcurity2 GPC-System with AGILENT infinity 1260 II hardware (Santa Clara, CA, USA) was used. The device uses a refractive index detector and runs on THF as a polar phase with a flow rate of 1 mL/min at 30 °C. The used column system consists of a PSS SDV analytical column (3 μm, 300 × 8.0 mm^2^, 1000 Å) with a PSS SDV analytical precolumn (3 μm, 50 × 8.0 mm^2^). Poly(methyl methacrylate) with masses ranging from 102 to 62,000 Da was used for calibration.

### 2.9. Optical Spectroscopy

The room-temperature steady-state PL spectra were recorded using an FS5 spectrofluorometer (Edinburgh Instruments, Livingston, UK), exciting at 370 nm. PLQYs were measured in an integrating sphere (FS5) with excitation from a xenon lamp (370 nm) under a nitrogen atmosphere. Neat films were fabricated by drop-casting toluene solutions of the compounds (100 µL, 2 mg mL^−1^) onto quartz substrates and then annealing at 50 °C for 15 min.

UV-vis absorption spectra were recorded on an Analytik Jena SPECORD 50 Plus (Jena, Germany). Before the measurement, the samples were dissolved in tetrahydrofuran and filled in glass cuvettes with a layer thickness of 0.1 cm.

### 2.10. Electronic Circular Dichroism (ECD) and Circularly Polarized Luminescence (CPL)

CD spectra of polymers were measured in dichloromethane solutions with a JASCO (Cremella, Italy) J-815 instrument. CPL measurements were performed using a home-built CPL spectrofluoropolarimeter (constructed with the help of the JASCO Europe, Cremella, Italy). The samples were excited using a 90° geometry with a 150 W LS Xenon ozone-free lamp. The samples were measured at ca. 5 × 10^−6^ m in CH_2_Cl_2_. Excitation of the samples was performed at 320 nm, and 5–9 scans accumulations were used.

### 2.11. Synthetic Procedures

Detailed synthetic procedures and analytical data for monomers and polymers can be found in the Supplementary Materials.

## 3. Results

**Figure 1:**
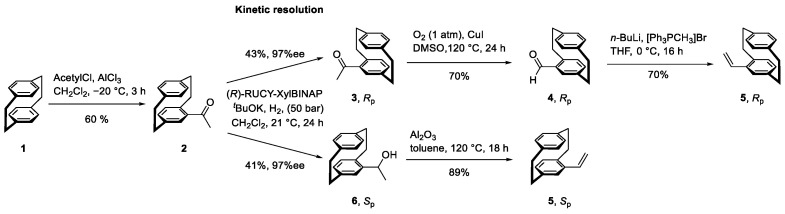
Synthesis of *R*_p_- and *S*_p_-vinyl[2.2]paracyclophane (**5**) via kinetic resolution using *R*-RUCY-XylBINAP.

To achieve the goal of chiral polymers, the first step was synthesizing the planar chiral monomers, namely R_p_- and S_p_-vinyl[2.2]paracyclophane (**5**). This was accomplished through a kinetic resolution previously reported by our group [23]. A three-to-four-step synthesis (Figure 1) yields the R_p_ enantiomer in 13% and the S_p_ enantiomer in 22% (using R-RUCY-XylBINAP, S-RUCY-XylBINAP will yield the reverse enantiomers), resulting in a combined 35% yield starting from PCP.

**Figure 2:**
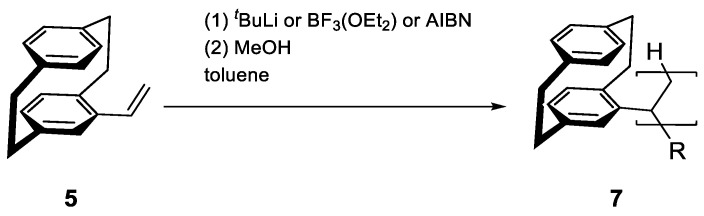
Homopolymerization of vinyl-PCP **5** in varying setups using anionic, cationic, and radical conditions in toluene to synthesize poly(vinyl[2.2]paracyclophane) (**7**).

With the chiral PCP monomer in hand, the next step was polymerizing chiral monomers (Figure 2). There are numerous possible methods for vinyl polymerization, the simplest being radical, anionic, and cationic.

These methods were first tested in 1994 for racemic vinyl-PCP by Iwatsuki et al. [22], who found that all three were applicable, with radical polymerization giving the highest molecular weight and anionic polymerization giving the highest conversion. Our tests (Table 1) with the chiral variant showed comparable results, with the ^1^H NMR proving the successful incorporation of PCP and showing broadened signals in the aromatic region as well as typical PCP bridge signals around 2.5–3.5 ppm (SI, Figures S4–S6). Furthermore, anionic polymerization using tert-butyllithium (^t^BuLi) yielded up to a 93% conversion rate and produced close to the expected decamer in mass. Although Iwatsuki’s work does not specify which BuLi was used, we additionally found that n- and sec-BuLi worked similarly but gave lower conversion. The results of cationic polymerization using BF_3_ etherate were also similar to Iwatsuki’s findings, with lower conversion (68%) and molecular masses significantly below expected levels. Radical polymerization with azobisisobutyronitrile (AIBN) showed slow and incomplete conversion, even after adding another 10 mol% initiator and extending the reaction time by 24 h. Only 18% conversion was achieved (9% after 24 h), but the number average molecular weight (M_n_) was close to that achieved with anionic polymerization (equivalent results were obtained with dibenzoyl peroxide as initiator). All results indicate a lower vinyl-PCP reactivity than styrene, which is also supported by its stability for multiple months without cooling or light exclusion. This may result from PCP being bulkier and sterically hindering polymerization.

We failed to observe higher molecular masses with lower initiator concentrations for both radical and anionic polymerization. In both cases, polymerization resulted in products that were insoluble in common organic solvents (toluene, THF, DCM, dimethylacetamide, hexafluoroisopropanol, EtOAc, acetone, MeCN, alcohols, and alkanes), suggesting the reaction of the soluble monomer but preventing analysis of molecular weight. To achieve soluble high-mass poly-PCP monomers, further functional groups, such as alkyl chains, could be tested in the future.

**Figure 3:**
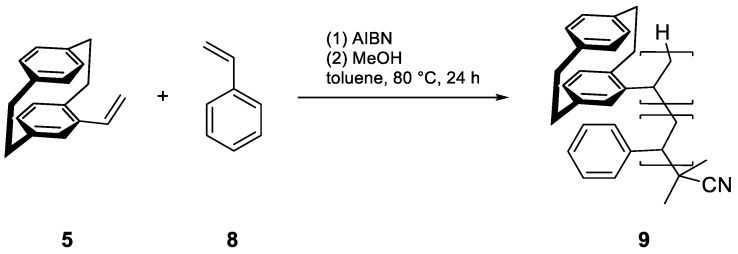
Radical copolymerization of vinyl-PCP and styrene using AIBN to yield poly(vinyl[2.2]paracyclophane-co-styrene) (**9**).

To improve the solubility and increase the molecular weight of the polymer using the given monomer, as well as to demonstrate that chiroptical properties can be maintained when combined with achiral monomers to broaden the range of applications, copolymerization with styrene was conducted in varying ratios (Figure 3). Styrene was chosen because of its ready availability and similarity to vinyl-PCP. Its extensively studied properties allow for a good comparison. The radical polymerization was chosen for ease of use, as it delivered more reliable results despite lower conversion than the anionic approach, which fails upon contact with even trace amounts of oxygen or moisture.

For copolymerization, PCP–styrene ratios of 1:1, 1:10, 1:20, and 1:50 were chosen, increasing the styrene share until no chiral properties could be detected in optical rotation measurements (Table 2). As expected, the addition of styrene greatly improved polymerization results, increasing conversions to 37–85% compared to a maximum of 18% for pure vinyl-PCP, while M_n_ rose to 2.56–6.02 kDa with M_w_ up to 13.9 kDa. ^1^H NMR spectral analysis verified that the monomer and ratio used in the final polymer were similar, indicating that styrene and vinyl-PCP were evenly consumed (SI, Figures S9 and S10, integral normalized to 8 for PCP bridge signal at 2.5–3.5 ppm). This supports the assumption that the low reactivity in polymerizations of pure vinyl-PCP stems from steric hindrance rather than generally low reactivity of the vinyl group itself. Even with higher weights, the solubility in organic solvents was vastly improved for the copolymers compared to the homopolymer. For example, in THF, solubility increased from <1 g/L for poly(vinyl[2.2]paracyclophane) with an M_n_ of approximately 1.8 kDa to >20 g/L for poly(vinyl[2.2]paracyclophane-co-styrene) with an M_n_ of 4.8 kDa and a ratio of 1:1.4. The specific rotation followed the expected trend, decreasing with an increasing share of achiral monomer. Due to the high starting values of the monomer at ±248 (deg*mL)/(g*dm) (compared to common natural chiral molecules: fructose = ±92.4 (deg*mL)/(g*dm) [25]; progesterone = 182 (deg*mL)/(g*dm) [26]; cholesterol = −37.5 (deg*mL)/(g*dm) [27]), even polymers with lower PCP ratios resulted in values potentially usable in chiroptical applications.

After these promising results showed that the polymers retain specific rotation even with considerable amounts of achiral copolymer, the next step was to explore further applications. To this end, we chose to combine the chiral vinyl-PCP with an emitter molecule to achieve CPL, which is useful in fields like sensing and optics [11].

The combination of an emitting and a non-emitting moiety offers several benefits. First, the non-emitting part of the molecule can act as a host material, preventing self-quenching effects by separating the emitter units. This would normally require a separate material, such as mCP (1,3-bis(N-carbazolyl)benzene) or DPEPO (bis[2-(diphenylphosphino)phenyl] ether oxide), when building a device but can now be integrated into the emitter polymer [28]. Second, the chirality of the non-emitter unit can influence the emitter polymer as a whole, yielding a CPL emitter without the need for a chiral center in the emitter itself.

**Figure 4:**
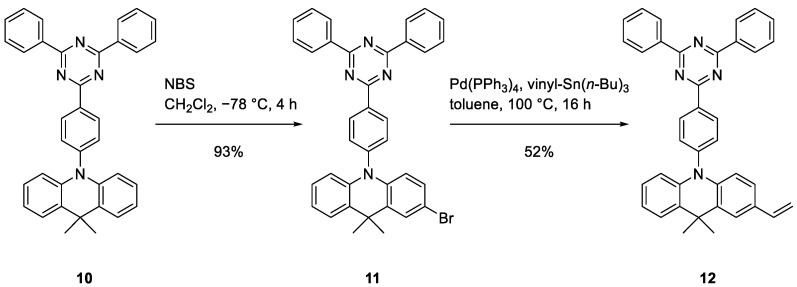
Synthesis of vinyl-DMAC-TRZ (**12**) by bromination with NBS and Stille coupling.

To copolymerize an emitter with vinyl-PCP, we required a polymerizable group, preferably another vinyl group. For this, we functionalized the well-known emitter DMAC-TRZ (**10**) in a straightforward two-step synthesis with an overall yield of 48%. First, a selective monobromination at the para position of the DMAC moiety was performed using N-bromosuccinimide (NBS) at a low temperature, followed by Stille coupling with tributyl-vinyl-stannane (Figure 4). This procedure can theoretically be applied to many common emitters, as all that is necessary is a position that can be halogenated.

**Figure 5:**
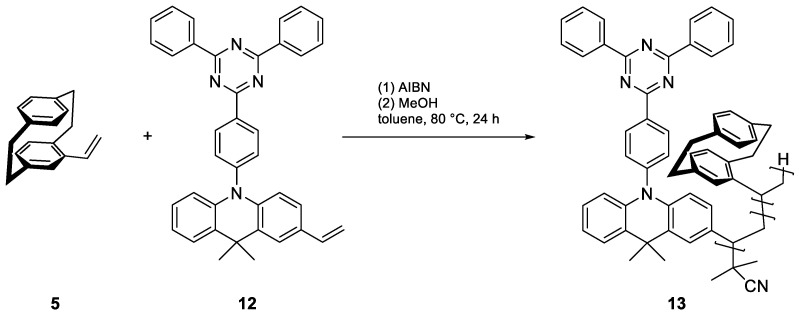
Radical copolymerization of vinyl-PCP (**5**) and vinyl-DMAC-TRZ (**12**) using AIBN yields poly(vinyl[2.2]paracyclophane-co-vinyl-DMAC-TRZ) (**13**).

Radical initiation with AIBN was chosen for copolymerization, as the more aggressive ^t^BuLi could also attack other positions in the emitter (Figure 5). Based on the above tests with styrene, it has been shown that small fractions as low as 2% of PCP can carry over the chirality. However, the amount of PCP must be significantly higher to separate the emitting moieties. To reflect this, PCP–emitter ratios of 10:1, 20:1, 50:1, and 100:1 were selected.

**Figure 6:**
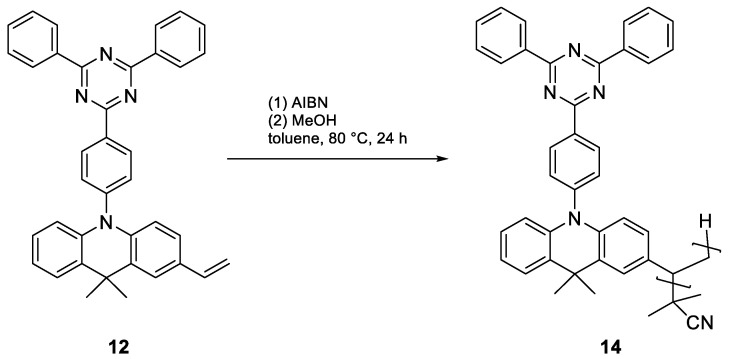
Radical homopolymerization of vinyl-DMAC-TRZ (**12**) using AIBN yields poly(vinyl-DMAC-TRZ) (**14**).

For comparison, pure vinyl-DMAC-TRZ (**12**) was also polymerized (Figure 6).

The radical polymerization in this setup had lower conversion and weight distributions (Table 3), probably due to the small scale of the polymerization and solubility issues, similar to the homopolymerization of vinyl-PCP. Additionally, the DMAC-TRZ monomer appeared to be incorporated preferentially compared to vinyl-PCP, resulting in 3–6 times more emitter moieties in the polymers than expected as estimated via NMR (SI, Figures S11–S16, integral normalized to 6 for the distinct DMAC-TRZ aromatic signal at 8.5–9.25 ppm). As discussed previously, the bulkiness of PCP hinders the continuous consumption of vinyl-PCP, a problem that the planar DMAC-TRZ moiety does not share, leading to higher emitter content in the polymer. With rising PCP content, the achieved molecular weight approaches the values observed for pure poly(vinyl-PCP), with the 50:1 and 100:1 ratios in the same range. The 20:1 result is somewhat of an outlier but within the range of fluctuations expected from the simplistic setup. While not optimized, these results demonstrate feasibility and can be improved with more sophisticated and specialized setups in future work.

**Figure 7:**
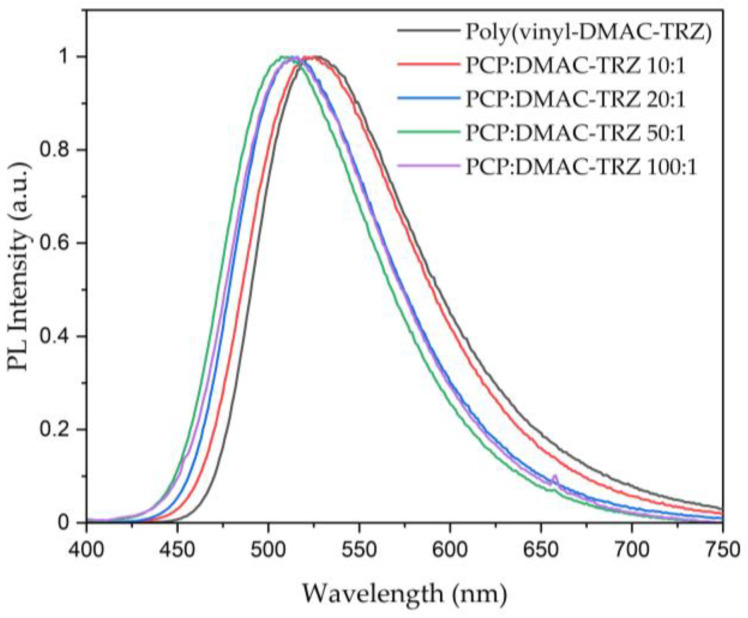
Photoluminescence spectra for poly(vinyl-PCP-co-vinyl-DMAC-TRZ) (**13**) and poly(vinyl-DMAC-TRZ) (**14**) upon excitation at 370 nm.

The resulting copolymers were tested for their photoluminescence (PL) properties, focusing on trends resulting from increasing PCP as a built-in host (Table 4). When excited at 370 nm under N_2_, increasing amounts of PCP led to a blue shift from 526 nm for the pure poly(vinyl-DMAC-TRZ) (**13**) to 510 nm for the 50:1-ratio polymer (Figure 7). With 513 nm, the 100:1-ratio polymer reverses the trend, which may be attributed to aggregation, also visible as precipitation from solutions and the hazy quality of the drop cast film. This trend holds true for the PLQY results: the values rose consistently from 26.8% for the pure emitter polymer in N_2_ to 47.2% for the 50:1-ratio polymer and then dropped to 38.0% for the 100:1-ratio polymer.

**Figure 8:**
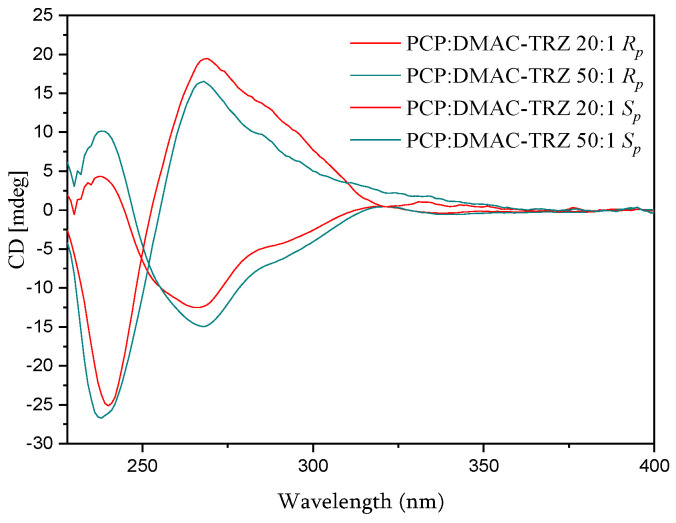
CD spectra of 20:1 and 50:1 polymers measured in CH_2_Cl_2_ at a molarity of ca. 5 × 10^−6^ M.

**Figure 9:**
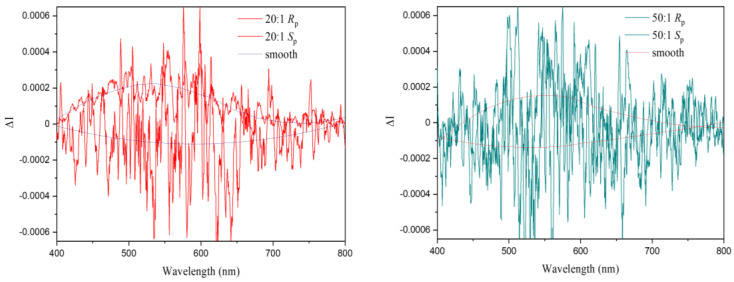
CPL measurements in 10^−6^ M DCM solution with excitation at 320 nm for poly(vinyl-PCP-co-vinyl-DMAC-TRZ) with 20:1 (**left**) and 50:1 (**right**) PCP–emitter ratio.

In combination with emission, the chirality of the polymers should make them CPL emitters. Measurements were performed with the 20:1 and 50:1 polymers, which showed the most promise in PL measurements. The chiroptical properties, i.e., CD and CPL, of the 20:1 and 50:1 polymers were thus examined. The CD spectra are displayed in Figure 8. They show clear mirror-image Cotton effects at 240 nm and 267 nm. Then, experimental measurements confirmed CPL activity for the 20:1 polymer (Figure 9, left) with a roughly mirror-imaged relationship for enantiomeric systems displaying similar molecular weights, as well as low dissymmetry factors (around +2 × 10^−4^ and −1 × 10^−4^) and noisy signals. The 50:1 polymer also indicated CPL, albeit with a very low signal-to-noise ratio (Figure 9, right). Future work should aim to find ways to increase CPL activity.

These results demonstrate that chiral vinyl-PCP monomers can successfully produce a CPL emitter from an achiral emitter. Combined with the above findings, chiral PCP polymers are a viable option for future application in functional materials that integrate chiral and optoelectronic properties.

## 4. Summary

In this work, we demonstrated using chiral vinyl-PCP as a monomer for homopolymerization and copolymerization with styrene. In the copolymerization process, we showed that the overall polymer still exhibits chirality even with just 2% chiral monomer, though at a lower intensity. Additionally, we developed a polymerizable vinyl emitter from DMAC-TRZ in a two-step synthesis with an overall yield of 48%, which was also successfully used in copolymerization with chiral vinyl-PCP. The resulting polymers exhibited CPL properties, with the PCP acting as both a host material and a source of chirality. The self-hosting effect peaks at 5–10% emitter content, achieving a PLQY of 47.2% PLQY in N_2_ compared to 26.8% for the pure emitter polymer without a host. In the future, optimization of the polymerization procedure to achieve more consistent results, tests with PCPs with better solubility, such as alkyl-substituted PCP, and implementation in actual applications, such as OLEDs, are in order.

## Figures and Tables

**Table 1:** Overview of conditions and results for the homopolymerization of chiral vinyl-PCP. M_n_ = number average molecular weight; M_w_ = weight average molecular weight; D = dispersity.

Enantiomer	Conditions	M_n_ [kDa]	M_w_ [kDa]	D	Conversion [%]
*S*_p_	10 mol% ^t^BuLi, toluene, 0 °C, 24 h	1.78	2.03	1.14	93
*R*_p_	10 mol% ^t^BuLi, toluene, 0 °C, 24 h	1.01	3.25	3.21	92
*S*_p_	1 mol% ^t^BuLi, toluene, 0 °C, 24 h	N/A	N/A	N/A	97 ^1^
*S*_p_	10 mol% BF_3_OEt_2_, toluene, 0 °C, 24 h	0.58	0.79	1.36	68
*S*_p_	10 mol% AIBN, toluene, 60 °C, 24 h	1.66	1.89	1.14	4
*R*_p_	10 mol% AIBN, toluene, 60 °C, 24 h	0.78	1.38	1.77	9
*S*_p_	2 × 10 mol% AIBN, toluene, 60 °C, 48 h	1.72	2.74	1.60	18
*S*_p_	1 mol% AIBN, bulk, 60 °C, 24 h	N/A	N/A	N/A	34 ^1^

^1^ Conversion for insoluble polymers are calculated by dividing mass of the resulting solid after washing steps by the mass of used monomers. Because of the limited analytics, no statement about purity can be made. GPC data is N/A for insoluble polymers since no data could be obtained.

**Table 2:** Overview of results for the copolymerization of *S*_p_-vinyl-PCP and styrene in varying ratios with 2 mol% AIBN after 24 h at 80 °C. M_n_ = number average molecular weight; M_w_ = weight average molecular weight, D = dispersity.

Sample	PCP–Styrene Ratio Determined by ^1^H NMR Spectroscopy	M_n_ [kDa]	M_w_ [kDa]	D	Conversion [%]	Specific Rotation α_D_^20^ [(deg*mL)/(g*dm)] Comparison: Vinyl Monomer ± 284
PCP–styrene 1:1	1:1.0	4.79	10.5	2.18	37	102.1
PCP–styrene 1:10	1:9.6	2.56	13.9	5.44	66	51.9
PCP–styrene 1:25	1:26.7	4.68	10.6	2.27	57	14.6
PCP–styrene 1:50	1:45.1	6.02	13.7	2.28	85	7.9

**Table 3:** Overview of results for the copolymerization of vinyl-PCP and vinyl-DMAC-TRZ in varying ratios with 2 mol% AIBN after 24 h at 80 °C. M_n_ = number average molecular weight; M_w_ = weight average molecular weight, D = dispersity.

Sample	PCP–DMAC-TRZ Ratio Determined by ^1^H NMR	M_n_ [kDa]	M_w_ [kDa]	D	Conversion [%]
Poly(vinyl-DMAC-TRZ) (**14**)	0:1	1.46	2.71	1.86	33
PCP–DMAC-TRZ 10:1 *R*_p_	3.7:1	1.17	6.57	5.63	20
PCP–DMAC-TRZ 20:1 *R*_p_	6.7:1	2.43	15.7	6.46	32
PCP–DMAC-TRZ 50:1 *R*_p_	11.1:1	0.89	3.98	4.47	30
PCP–DMAC-TRZ 100:1 *R*_p_	18.1:1	0.51	1.96	3.85	23
PCP–DMAC-TRZ 20:1 *S*_p_	7.8:1	0.73	2.19	2.99	56
PCP–DMAC-TRZ 50:1 *S*_p_	17.7:1	0.76	2.27	3.00	28

**Table 4:** Results of PL measurements as well as emission lifetimes for poly(vinyl-DMAC-TRZ) and the varying ratios of copolymer *R*_p_-poly(vinyl-PCP-co-vinyl-DMAC-TRZ).

Sample	λ_max_ at 370 nm Excitation (nm)	PLQY in N_2_ (%)
Poly(vinyl-DMAC-TRZ) (**14**)	526	26.8
PCP–DMAC-TRZ 10:1 *R*_p_	525	29.1
PCP–DMAC-TRZ 20:1 *R*_p_	513	40.8
PCP–DMAC-TRZ 50:1 *R*_p_	510	47.2
PCP–DMAC-TRZ 100:1 *R*_p_	513	38.0

## Data Availability

The data presented in this study are openly available in the repository Chemotion (https://www.chemotion-repository.net/ accessed on 24 February 2025) at https://dx.doi.org/10.14272/collection/HT_2024-05-03 accessed on 24 February 2025.

## Supplementary Materials

The following supporting information can be downloaded at https://www.mdpi.com/article/10.3390/polym17081070/s1, Synthetic Procedures Monomers, Synthetic Procedures Polymers. Figure S1: ^1^H (top) and ^13^C NMR (bottom) of **10**; Figure S2: ^1^H (top) and ^13^C NMR (bottom) of **11**; Figure S3: ^1^H (top) and ^13^C NMR (bottom) of **12**; Figure S4: ^1^H NMR of **7** synthesized via anionic polymerization; Figure S5: ^1^H NMR of **7** synthesized via radical polymerization; Figure S6: ^1^H NMR of **7** synthesized via cationic polymerization; Figure S7: ^1^H NMR of **9** synthesized using a 1:1 ratio; Figure S8: ^1^H NMR of **9** synthesized using a 1:10 ratio; Figure S9: ^1^H NMR of **9** synthesized using a 1:25 ratio; Figure S10: ^1^H NMR of **9** synthesized using a 1:50 ratio; Figure S11: ^1^H NMR of R_p_-**13** synthesized using a 10:1 ratio; Figure S12: ^1^H NMR of R_p_-**13** synthesized using a 20:1 ratio; Figure S13: ^1^H NMR of R_p_-**13** synthesized using a 50:1 ratio; Figure S14: ^1^H NMR of R_p_-**13** synthesized using a 100:1 ratio; Figure S15: ^1^H NMR of S_p_-**13** synthesized using a 20:1 ratio; Figure S16: ^1^H NMR of S_p_-**13** synthesized using a 50:1 ratio; Figure S17: ^1^H NMR of 14.
