# Supplementary material for: Chiral Polymers Based on Vinyl[2.2]paracyclophane and Their Application as CPL Emitters
Source: Polymers (Basel). 2025 Apr 16;17(8):1070. doi: 10.3390/polym17081070 (PMC12030565; doi:10.3390/polym17081070)
Supplement: Supplementary file 1 [file polymers-17-01070-s001.zip › polymers-3526253-supplementary.pdf]

polymers

MDPI

Supplementary Materials

Chiral Polymers Based on Vinyl[2.2]paracyclophane and Their Application as CPL Emitters

Henrik Tappert [1], Emma V. Puttock [1,2], Jhon Sebastian Oviedo Ortiz [3], Eli Zysman-Colman [4], Jeanne Crassous [3] and Stefan Bräse [1,2,*]

[1] Institute of Organic Chemistry (IOC), Karlsruhe Institute of Technology (KIT), Kaiserstraße 12, 76131 Karlsruhe, Germany; henrik.tappert@kit.edu (H.T.); emma.puttock@kit.edu (E.V.P.)
[2] Institute of Biological and Chemical Systems-Functional Molecular Systems (IBCS-FMS), Karlsruhe Institute of Technology (KIT), Kaiserstraße 12, 76131 Karlsruhe, Germany
[3] Institut des Sciences Chimiques de Rennes, Univ Rennes, Unité Mixte de Recherche (UMR) Centre National de la Recherche Scientifique (CNRS) 6226, Campus de Beaulieu, 35042 Rennes, CEDEX, France; jhon-sebastian.ortiz@univ-rennes.fr (J.S.O.O.); jeanne.crassous@univ-rennes.fr (J.C.)
[4] Organic Semiconductor Centre, EaStCHEM School of Chemistry, University of St Andrews, Fife, St Andrews KY16 9ST, UK; eli.zysman-colman@st-andrews.ac.uk
* Correspondence: braese@kit.edu

1. Synthetic Procedures Monomers

The molecules **2-6** were synthesized following the procedures of Zippel et al. [1] The analytics matched the literature.

10-[4-(4,6-Diphenyl-1,3,5-triazin-2-yl)phenyl]-9,9-dimethyl-acridine (DMAC-TRZ) (10)

A vial was charged with 9,9-dimethyl-10H-acridine (750 mg, 3.58 mmol, 1.00 equiv.), 2-(4-bromophenyl)-4,6-diphenyl-1,3,5-triazine (1.53 g, 3.94 mmol, 1.10 equiv.) and KO^{t}Bu (1.21 g, 10.8 mmol, 3.00 equiv.). It was evacuated and flushed with argon three times. Anhydrous toluene (50.0 mL) was added, then heated to 100 °C and stirred for 16 h. After cooling to 21 °C, the reaction mixture was washed with 3 x 50 mL brine. The organic layer was dried over Na_2SO_4, and the solvent was removed under reduced pressure. The obtained crude product was purified via column chromatography on silica gel using pentane/dichloromethane 10:1 as eluent to yield 10-[4-(4,6-diphenyl-1,3,5-triazin-2-yl)phenyl]-9,9-dimethyl-acridine (1.30 g, 2.52 mmol, 70% yield) as a yellow solid.

R_f = 0.57 (pentane:CH_2Cl_2 = 4:1).

^{1}H NMR (400 MHz, Chloroform-d [7.26 ppm], ppm) δ = 9.05–9.02 (m, 2H, CH_{Ar}), 8.84–8.82 (m, 4H, CH_{Ar}), 7.68–7.56 (m, 8H, CH_{Ar}), 7.52–7.49 (m, 2H, CH_{Ar}), 7.03–6.95 (m, 4H, CH_{Ar}), 6.40 (dt, *J* = 7.8 Hz, *J* = 1.3 Hz, 2H, CH_{Ar}), 1.74 (s, 6H, CH_3);

^{13}C NMR (100 MHz, Chloroform-d [77.16 ppm], ppm) δ = 171.9 (C_q, 2C), 171.1 (C_q), 145.4 (C_q), 140.6 (C_q, 2C), 136.1 (C_q), 136.1 (C_q, 2C), 132.7 (+, CH, 2C), 131.6 (+, CH, 2C), 131.5 (+, CH, 2C), 130.3 (C_q, 2C), 129.0 (+, CH, 4C), 128.7 (+, CH, 4C), 126.5 (+, CH, 2C), 125.4 (+, CH, 2C), 120.9 (+, CH, 2C), 114.2 (+, CH, 2C), 36.1 (C_q), 31.3 (+, CH_3, 2C);

MS (EI, 70 eV, 60 °C), m/z (%): 517 (28) $[M+H]^+$, 516 (16) $[M]^+$, 501 (34), 307 (30), 155 (27), 154 (100), 138 (32), 137 (60), 136 (63). HRMS (EI): m/z = calcd for $C_{36}H_{29}N_4$ $[M+H]^+$: 517.2387; found 517.2385;

IR (ATR, ṽ) = 3070 (vw), 3031 (vw), 2962 (vw), 2955 (vw), 1588 (m), 1511 (vs), 1475 (vs), 1465 (s), 1445 (vs), 1408 (m), 1363 (vs), 1326 (vs), 1269 (vs), 1170 (m), 1159 (w), 1146 (w), 1126 (w), 1113 (w), 1098 (w), 1089 (w), 1067 (w), 1047 (w), 1024 (w), 1017 (w), 1001

(w), 989 (w), 975 (w), 926 (w), 836 (m), 769 (m), 739 (vs), 694 (s), 681 (vs), 666 (m), 645 (m), 636 (w), 622 (m), 605 (w), 578 (w), 551 (w), 518 (m), 477 (w), 450 (w), 409 (w) cm^{-1}.

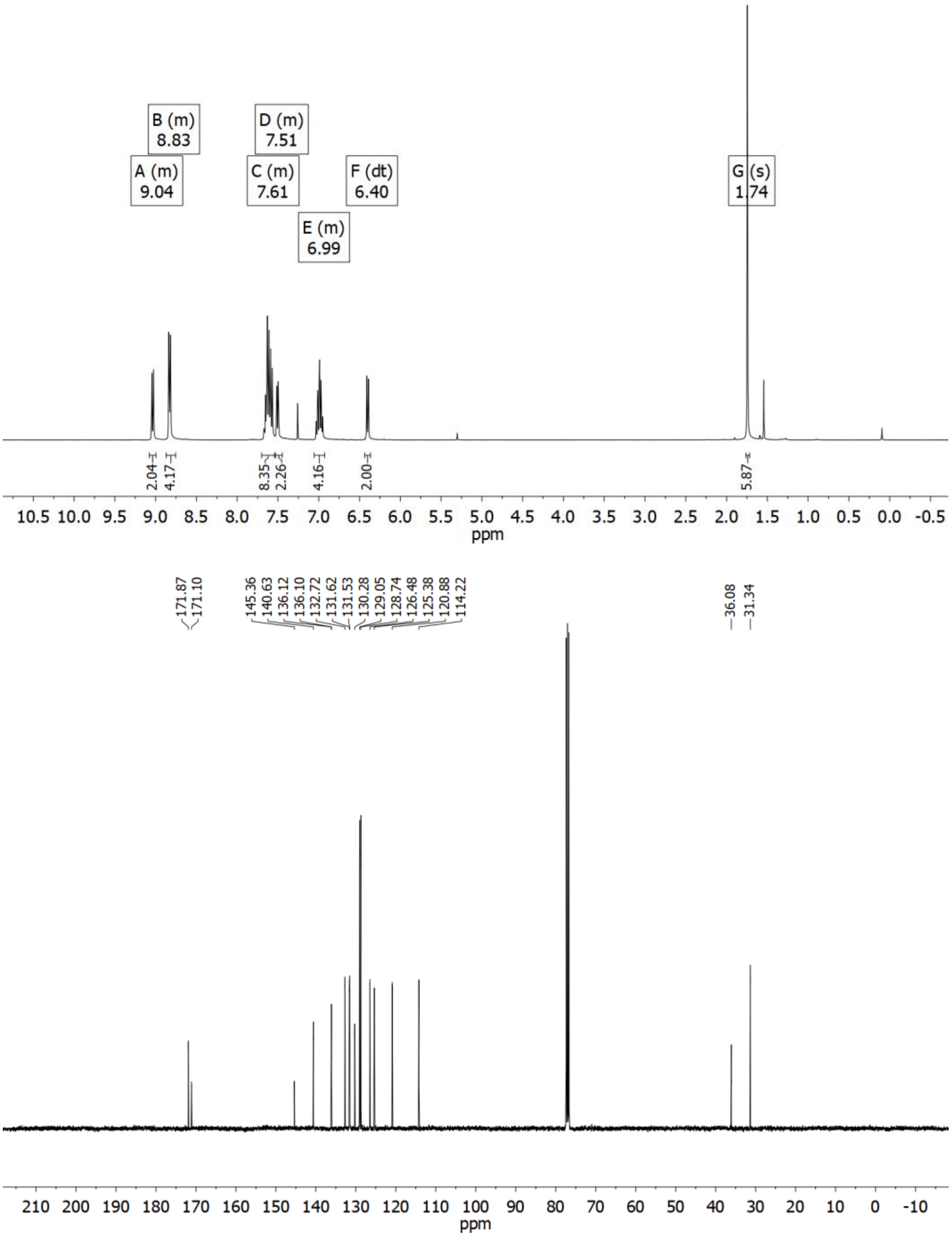

Figure S1. ^{1}H (top) and ^{13}C NMR (bottom)of **10**.

Additional information on the chemical synthesis is available via the Chemotion repository: https://doi.org/10.14272/reaction/SA-FUHFF-UHFFFADPSC-IVXBGKPGZO-UHFFFADPSC-NUHFF-NUHFF-NUHFF-ZZZ (accessed on 03.04.2025).

Additional information on the analysis of the target compound is available via the Chemotion repository: https://doi.org/10.14272/IVXBGKPGZOETEW-UHFFFAOYSA-N.1 (accessed on 03.04.2025).

2-Bromo-10-(4-(4,6-diphenyl-1,3,5-triazin-2-yl)phenyl)-9,9-dimethyl-9,10-dihydroacridine (11)

1-Bromopyrrolidine-2,5-dione (543 mg, 3.05 mmol, 1.05 equiv.) was added portionwise to a dry-ice-cooled solution of 10-[4-(4,6-diphenyl-1,3,5-triazin-2-yl)phenyl]-9,9-

dimethylacridine (1.50 g, 2.90 mmol, 1.00 equiv.) in 75.0 mL of dichloromethane while stirring over 2 h. The solution was stirred for another hour and then warmed to 21 °C. It was then washed with water (3 x 100 mL), dried over Na_2SO_4 and subjected to column chromatography on silica with pentane/dichloromethane 10:1 as eluent to yield the product 2-bromo-10-(4-(4,6-diphenyl-1,3,5-triazin-2-yl)phenyl)-9,9-dimethyl-9,10-dihydroacridine (1.61 g, 2.71 mmol, 93% yield) as a yellow solid.

R_f = 0.51 (pentane:CH_2Cl_2 = 4:1).

^{1}H NMR (400 MHz, Chloroform-d [7.26 ppm], ppm) δ = 9.05–9.01 (m, 2H, CH_{Ar}), 8.85–8.79 (m, 4H, CH_{Ar}), 7.67–7.59 (m, 6H, CH_{Ar}), 7.56 (d, *J* = 2.3 Hz, 1H, CH_{Ar}), 7.56–7.52 (m, 2H, CH_{Ar}), 7.50–7.47 (m, 1H, CH_{Ar}), 7.08 (dd, *J* = 8.8 Hz, *J* = 2.3 Hz, 1H, CH_{Ar}), 7.03–6.96 (m, 2H, CH_{Ar}), 6.41–6.35 (m, 1H, CH_{Ar}), 6.24 (d, *J* = 8.8 Hz, 1H, CH_{Ar}), 1.70 (s, 6H, CH_3);

^{13}C NMR (100 MHz, Chloroform-d [77.16 ppm], ppm) δ = 171.9 (C_q, 2C), 171.0 (C_q), 144.8 (C_q), 140.2 (C_q), 139.8 (C_q), 136.4 (C_q), 136.0 (C_q, 2C), 132.8 (+, CH, 2C), 132.4 (C_q), 131.7 (+, CH, 2C), 131.4 (+, CH, 2C), 129.7 (C_q), 129.2 (+, CH), 129.0 (+, CH, 4C), 128.7 (+, CH, 4C), 128.2 (+, CH), 126.7 (+, CH), 125.3 (+, CH), 121.2 (+, CH), 115.8 (+, CH), 114.3 (+, CH), 113.2 (C_q), 36.2 (C_q), 31.2 (+, CH3, 2C);

MS (FAB, 3-NBA), m/z (%): 595/597 (6/6) $[M+H]^+$, 594/596 (5/4) [M]+, 307 (35), 155 (30), 154 (100), 138 (34), 137 (66), 136 (67). HRMS (FAB, matrix NBA): m/z = calcd for $C_{36}H_{28}N_4{}^{79}Br$ $[M+H]^+$: 595.1492; found 595.1491;

IR (ATR, ṽ) = 3067 (w), 3034 (w), 2959 (w), 2918 (w), 2859 (w), 1588 (m), 1521 (vs), 1500 (s), 1476 (vs), 1460 (s), 1446 (s), 1408 (m), 1366 (vs), 1332 (s), 1316 (s), 1285 (m), 1269 (m), 1230 (w), 1215 (w), 1169 (w), 1146 (w), 1123 (w), 1096 (w), 1084 (w), 1067 (w), 1048 (w), 1026 (w), 1017 (w), 1001 (w), 932 (w), 878 (w), 839 (m), 802 (m), 771 (s), 742 (vs), 690 (vs), 680 (s), 670 (w), 656 (w), 646 (m), 635 (w), 606 (w), 565 (w), 551 (w), 533 (s), 517 (m), 484 (w), 470 (w), 446 (w), 415 (w), 402 (w), 394 (w), 377 (w) cm^{-1}.

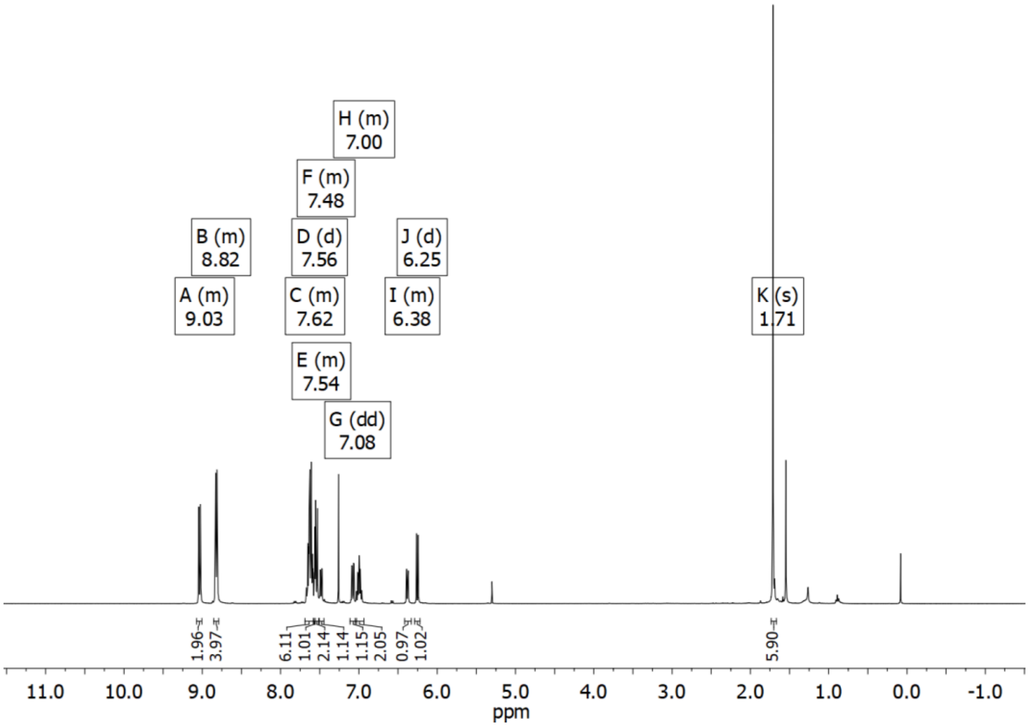

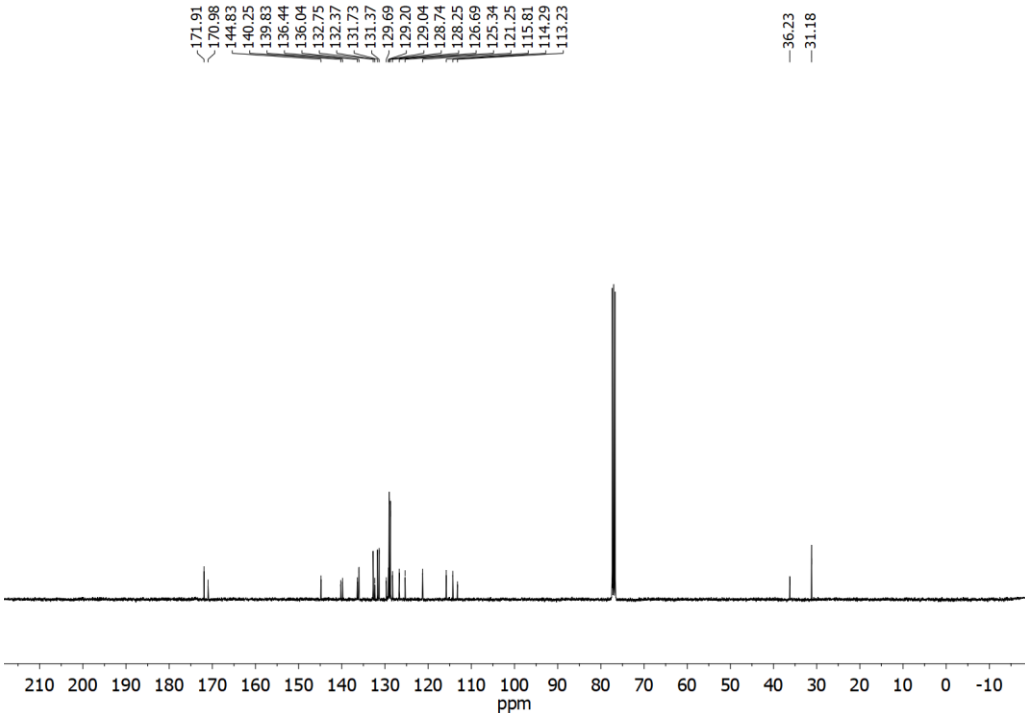

Figure S2. ^{1}H (top) and ^{13}C NMR (bottom)of **11**.

Additional information on the chemical synthesis is available via the Chemotion repository: https://doi.org/10.14272/reaction/SA-FUHFF-UHFFFADPSC-QDIRQRCGOT-UHFFFADPSC-NUHFF-NUHFF-NUHFF-ZZZ (accessed on 03.04.2025).

Additional information on the analysis of the target compound is available via the Chemotion repository: https://doi.org/10.14272/QDIRQRCGOTXLOJ-UHFFFAOYSA-N.1 (accessed on 03.04.2025).

10-(4-(4,6-Diphenyl-1,3,5-triazin-2-yl)phenyl)-9,9-dimethyl-2-vinyl-9,10-dihydroacridine (12)

Tetrakis(triphenylphosphine)palladium(0) (291 mg, 252 μmol, 0.100 equiv.) and 2-bromo-10-(4-(4,6-diphenyl-1,3,5-triazin-2-yl)phenyl)-9,9-dimethyl-9,10-dihydroacridine (1.50 g, 2.52 mmol, 1.00 equiv.) were added together under argon and dissolved in 75 mL of dry toluene. After stirring for 15 min, tributyl(vinyl)stannane (1.20 g, 1.10 mL, 3.78 mmol, 1.50 equiv.) was added dropwise. The mixture was stirred for 12 h at 100 °C. The mixture was cooled to 21 °C, poured into 300 mL of saturated KF_{aq} solution, and extracted with 3 x 50 mL of DCM. The organic extracts were washed with 2 x 100 mL of brine and then dried over Na_2SO_4. After evaporating the solvent, the residue was purified by column chromatography on silica using pentane/DCM 10:1 as eluent to yield 10-(4-(4,6-diphenyl-1,3,5-triazin-2-yl)phenyl)-9,9-dimethyl-2-vinyl-9,10-dihydroacridine (712 mg, 93% purity, 1.22 mmol, 48% yield) as a yellow solid.

R_f = 0.44 (pentane:CH_2Cl_2 = 4:1).

^{1}H NMR (400 MHz, Chloroform-d [7.26 ppm], ppm) δ = 9.05–9.03 (m, 2H, CH_{Ar}), 8.83 (m, 4H, CH_{Ar}), 7.68–7.49 (m, 10H, CH_{Ar}), 7.08 (dd, *J* = 8.6 Hz, *J* = 2.0 Hz, 1H, CH_{Ar}), 7.03–6.95 (m, 2H, CH_{Ar}), 6.69 (dd, *J* = 17.5 Hz, J = 10.9 Hz, 1H, vinyl-H), 6.39 (m, 1H, CH_{Ar}), 6.35 (d, *J* = 8.5 Hz, 1H, CH_{Ar}), 5.62 (dd, *J* = 17.5 Hz, *J* = 1.0 Hz, 1H, vinyl-H), 5.12 (dd, *J* = 10.8 Hz, *J* = 1.0 Hz, 1H, vinyl-H), 1.77–1.74 (m, 6H, CH_3);

^{13}C NMR (100 MHz, Chloroform-d [77.16 ppm], ppm) δ = 171.9 (C_q, 2C), 171.0 (C_q), 145.2 (C_q), 140.3 (C_q), 140.3 (C_q), 136.6 (+, CH, vinyl-CH), 136.2 (C_q), 136.1 (C_q, 2C), 132.7 (+, CH, 2C), 131.6 (+, CH, 2C), 131.5 (+, CH, 2C), 130.4 (C_q), 130.1 (C_q), 130.1 (C_q), 129.0 (+, CH, 4C), 128.8 (+, CH, 4C), 126.5 (+, CH), 125.5 (+, CH), 124.3 (+, CH), 123.7 (+, CH), 121.0 (+, CH), 114.3 (+, CH), 114.2 (+, CH), 110.9 (–, CH_2, vinyl-CH_2), 36.1 (C_q), 31.6 (+, CH_3, 2C);

MS (FAB, 3-NBA), m/z (%): 543 (2) [M+H]$^+$, 307 (36), 155 (29), 154 (100),138 (34), 137 (66), 136 (64). HRMS (FAB, matrix NBA): m/z = calcd for $C_{38}H_{31}N_4$ [M+H]$^+$: 543.2543 ; found 543.2541;

IR (ATR, ṽ) = 1588 (m), 1520 (vs), 1482 (vs), 1463 (m), 1445 (vs), 1408 (m), 1397 (w), 1366 (vs), 1333 (vs), 1293 (s), 1271 (s), 1232 (w), 1228 (w), 1197 (w), 1176 (m), 1169 (m), 1146 (m), 1125 (w), 1088 (w), 1067 (w), 1050 (w), 1024 (w), 1016 (m), 993 (w), 975 (w), 931 (w), 898 (m), 881 (w), 843 (m), 830 (w), 815 (w), 803 (w), 769 (s), 751 (vs), 741 (vs), 690 (vs), 680 (s), 663 (m), 646 (s), 635 (m), 608 (w), 596 (w), 586 (w), 564 (w), 526 (m), 517 (m), 472 (w), 453 (w), 429 (w), 401 (w) cm^{-1}.

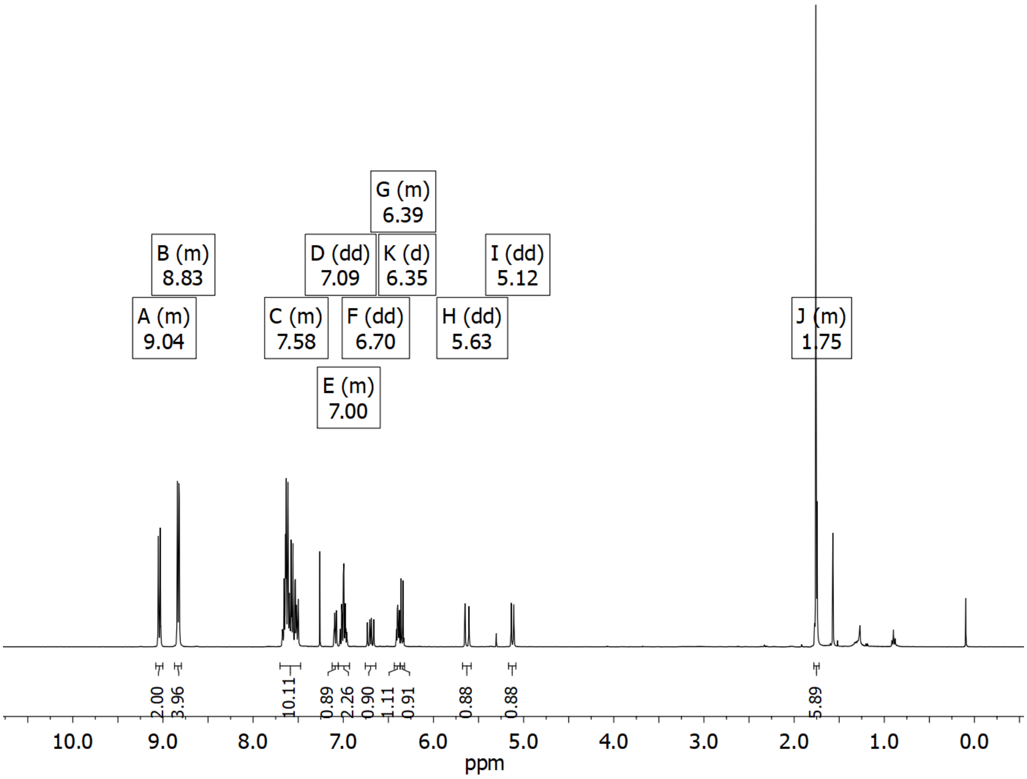

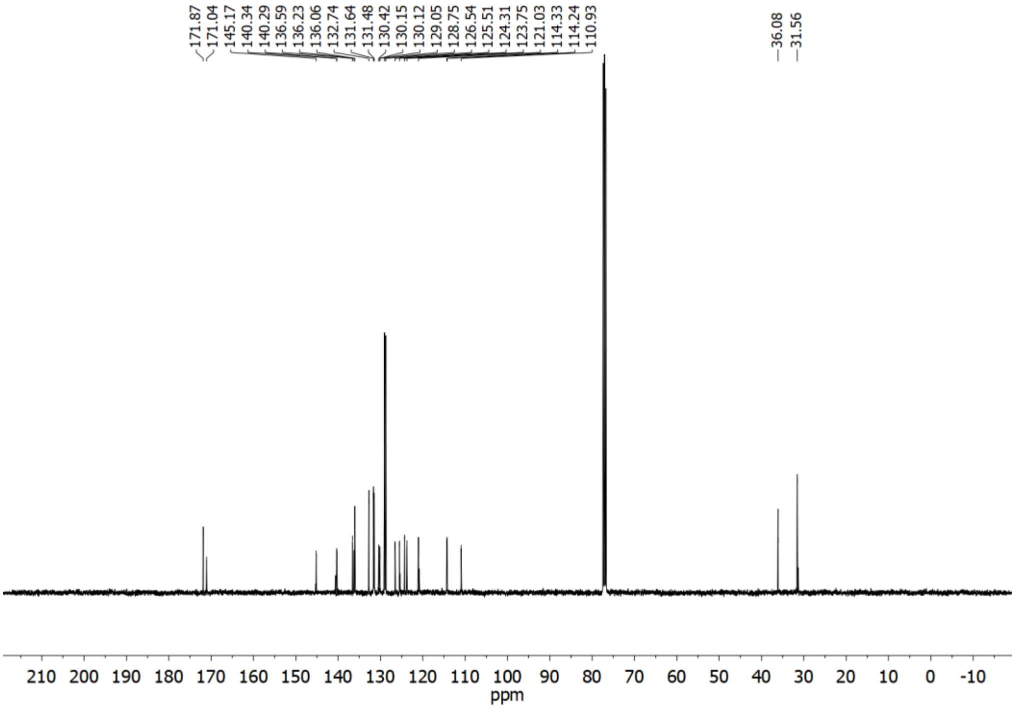

Figure S3. ^{1}H (top) and ^{13}C NMR (bottom)of **12**.

Additional information on the chemical synthesis is available via the Chemotion repository: https://doi.org/10.14272/reaction/SA-FUHFF-UHFFFADPSC-PJYLHMAQYD-UHFFFADPSC-NUHFF-NUHFF-NUHFF-ZZZ (accessed on 03.04.2025).

Additional information on the analysis of the target compound is available via the Chemotion repository: https://doi.org/10.14272/PJYLHMAQYDQPAM-UHFFFAOYSA-N.1 (accessed on 03.04.2025).

2. Synthetic Procedures Polymers

2.1. General Procedure Homopolymerization Vinyl-PCP (7)

Anionic Polymerization

The polymerization reactor was immersed in a thermostated iPrOH bath to maintain the reaction temperature at 0 °C. Under argon atmosphere, the initiator tert-butyllithium (0.0100 – 0.100 equiv.) was added to 4-vinyl[2.2]paracyclophane (250 mg, 1.07 mmol, 1.00 equiv.) in toluene (0.2 mL) to start the reaction. After 24 h, the reaction was terminated by precipitating from 10 mL of methanol, and the product was collected by centrifugation. The solid was redissolved in 1.5 mL of DCM and precipitated from 10 mL of methanol to yield poly(vinyl[2.2]paracyclophane).

^{1}H NMR (400 MHz, Dichloromethane-d_2 [5.32 ppm], ppm) δ = 6.89–5.78 (m, 7H), 3.65–2.46 (m, 8H), 1.34–1.23 (m, 2H), 0.98–0.80 (m, 1H). Solvents: 7.24 + 7.15 + 2.34 ppm (toluene), 1.56 ppm (water).

GPC S_p: M_n: 1.781E^{+3} g/mol; M_w: 2.036E^{+3} g/mol; D: 1.143E^{+0};

GPC R_p: M_n: 1.011E^{+3} g/mol; M_w: 3.245E^{+3} g/mol; D: 3.211E^{+0}.

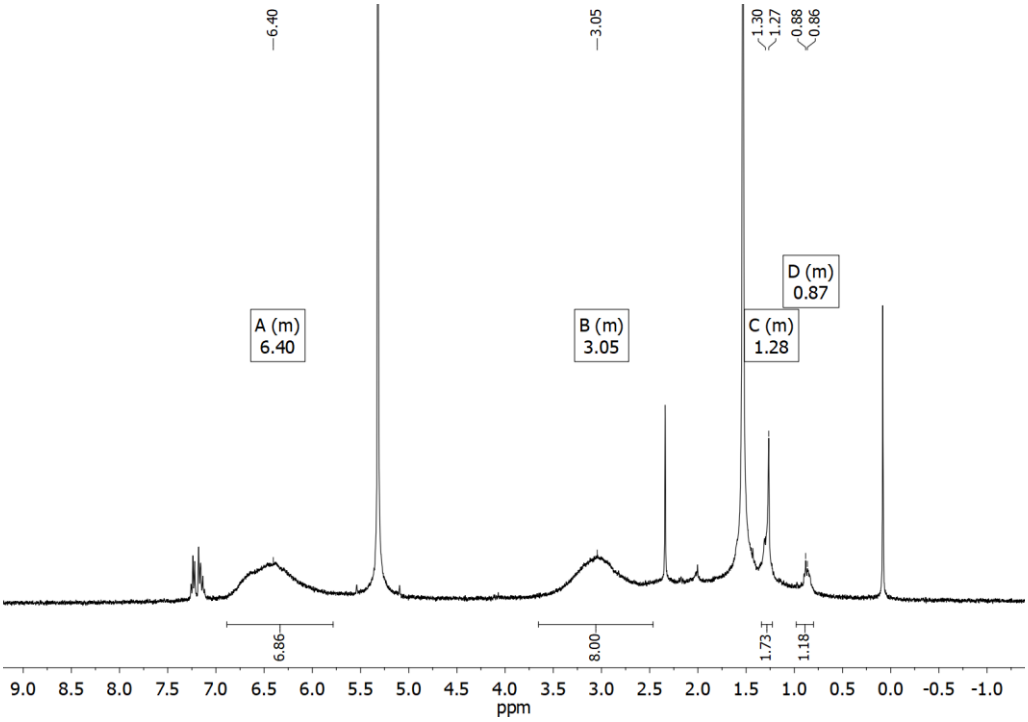

Figure S4. ^{1}H NMR of **7** synthesized via anionic polymerization.

Additional information on the chemical synthesis is available via the Chemotion repository: https://doi.org/10.14272/reaction/SA-FUHFF-UHFFFADPSC-UHFFFADPSC-UHFFFADPSC-NUHFF-NUHFF-NUHFF-ZAZ.10 (accessed on 03.04.2025).

Additional information on the analysis of the target compound is available via the Chemotion repository: https://doi.org/0.14272/DUMMY.9 (accessed on 03.04.2025).

Radical Polymerization

Under an argon atmosphere, the initiator AIBN (0.0100 – 0.200 equiv.) was added to R_p- or S_p-4-vinyl[2.2]paracyclophane (250 mg, 1.07 mmol, 1.00 equiv.) in dry toluene (1 mL) to start the reaction and heated to 60 °C for 24 h. The reaction was terminated by precipitating from 10 mL of methanol, and the product was collected by centrifugation. The solid was redissolved in 1.5 mL of DCM and precipitated from 10 mL of methanol to yield poly(vinyl[2.2]paracyclophane).

^{1}H NMR (400 MHz, Dichloromethane-d_2 [5.32 ppm], ppm) δ = 6.94–5.76 (m, 7H), 3.67–2.48 (m, 8H), 2.16–0.69 (m, 3H).

GPC S_p: M_n: 1.665E^{+3} g/mol; M_w: 1.898E^{+3} g/mol; D: 1.140E^{+0};

GPC R_p: M_n: 7.784E^{+2} g/mol; M_w: 1.379E^{+3} g/mol; D: 1.772E^{+0};

GPC S_p, double run: M_n: 1.716E^{+3} g/mol; M_w: 2.744E^{+3} g/mol; D: 1.599E^{+0}.

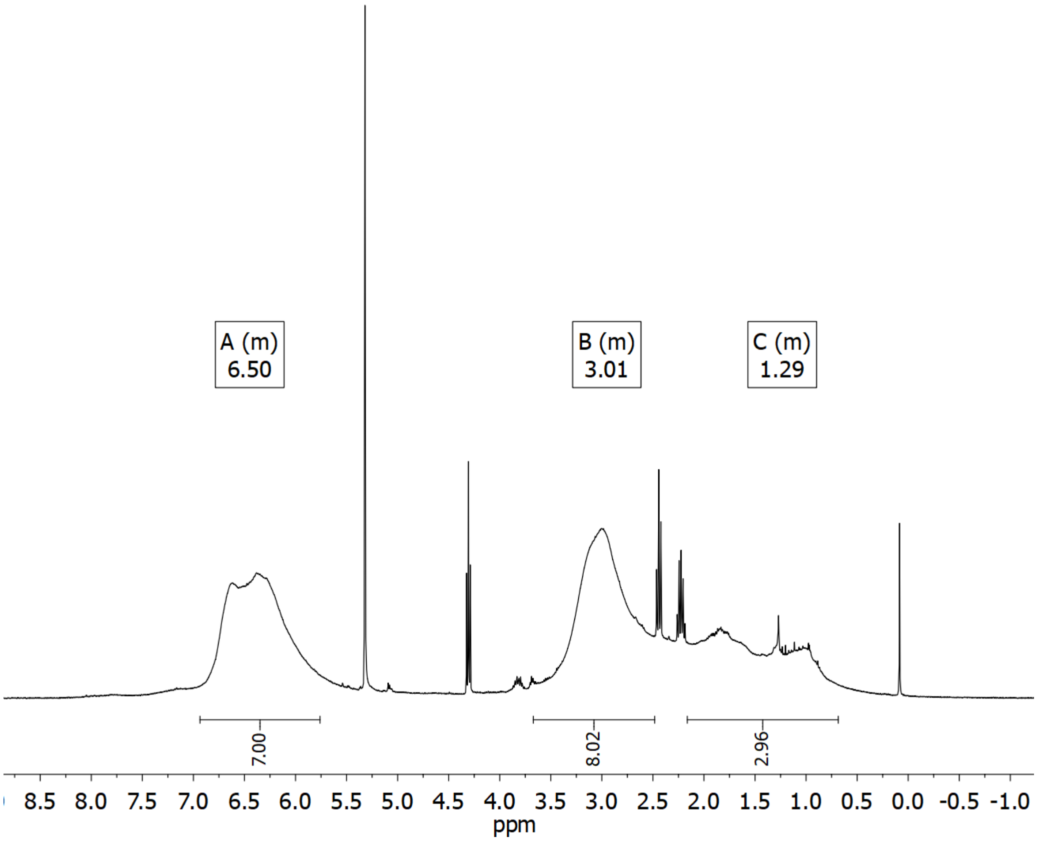

Figure S5. ^{1}H NMR of **7** synthesized via radical polymerization.

Additional information on the chemical synthesis is available via the Chemotion repository: https://doi.org/10.14272/reaction/SA-FUHFF-UHFFFADPSC-MVZQZJZNEJ-UHFFFADPSC-NUHFF-NUHFF-NUHFF-ZZZ (accessed on 03.04.2025).

Additional information on the analysis of the target compound is available via the Chemotion repository: https://doi.org/10.14272/MVZQZJZNEJDERU-UHFFFAOYSA-N.1 (accessed on 03.04.2025).

Cationic Polymerization

The polymerization reactor was immersed in a thermostated iPrOH bath to maintain the reaction temperature at 0 °C. Under argon atmosphere, the initiator $BF_3 \cdot OEt_2$ (31.5 mg, 28.2 µL, 213 µmol, 0.100 equiv.) was added to S_p-4-vinyl[2.2]paracyclophane (500 mg, 2.13 mmol, 1.00 equiv.) in dry toluene (1 mL) to start the reaction. After 24 h, the reaction was terminated by precipitating from 10 mL of methanol, and the product was collected by centrifugation. The solid was redissolved in 1.5 mL of DCM and precipitated from 10 mL of methanol to yield S_p-poly(vinyl[2.2]paracyclophane).

^{1}H NMR (400 MHz, Chloroform-d [7.26 ppm], ppm) δ = 6.86–5.81 (m, 7H), 3.46–2.41 (m, 8H), 1.49–0.54 (m, 3H).

GPC: M_n: 5.820E^{+2} g/mol; M_w: 7.890E^{+2} g/mol; D: 1.356E^{+0}.

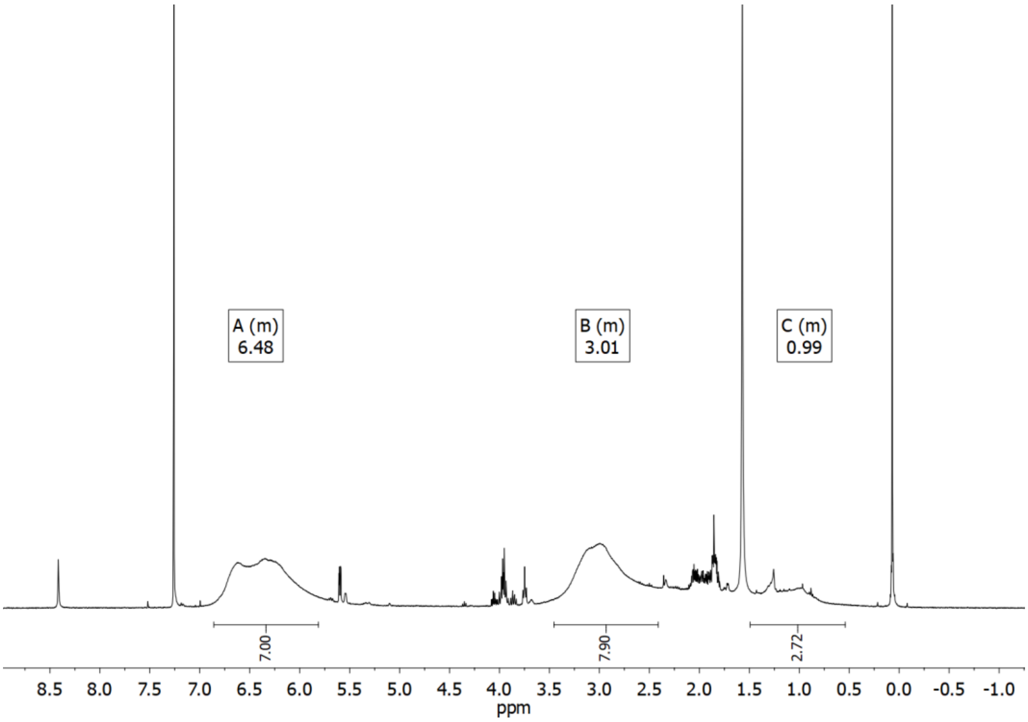

Figure S6. ^{1}H NMR of **7** synthesized via cationic polymerization.

Additional information on the chemical synthesis is available via the Chemotion repository: https://doi.org/10.14272/reaction/SA-FUHFF-UHFFFADPSC-OJVMIRUKBR-UHFFFADPSC-NUHFF-NUHFF-NUHFF-ZZZ (accessed on 03.04.2025).

Additional information on the analysis of the target compound is available via the Chemotion repository: https://doi.org/10.14272/OJVMIRUKBRPGIQ-UHFFFAOYSA-N.1 (accessed on 03.04.2025).

2.2. General Procedure Copolymerization Vinyl-PCP + Styrene (9)

In a Schlenck tube, 0.02 eq/monomer of the initiator AIBN was added to styrene (1.00 – 50.0 equiv.), and S_p-4-vinyl[2.2]paracyclophane (25.0 mg, 107 µmol, 1.00 equiv.) in 0.250 mL of dry degassed toluene and the reaction heated to 80 °C to start the reaction. The reaction was cooled and terminated by precipitation by adding 5 mL of methanol after 24 h. The product was collected by centrifugation and redissolved in 0.250 mL of DCM. Afterward, it was precipitated by adding 5 mL of methanol, collected by centrifugation, and dried in a vacuum to yield S_p-poly(styrene-co-vinyl[2.2]paracyclophane).

PCP:styrene 1:1:

^{1}H NMR (400 MHz, Dichloromethane-d_2 [5.32 ppm], ppm) δ = 7.56-6.82 (m, 4H, CH_{Ar}), 6.67–5.77 (m, 8H, CH_{Ar}), 3.33–2.26 (m, 8H, ethylene-bridge-CH_2), 2.25–1.49 (m, 5H, alkyl-H), 1.37–1.16 (m, 1H, alkyl-H);

GPC: M_n: 4.790*E^{+3} g/mol; M_w: 1.046*E^{+4} g/mol; D: 2.183*E^{+0};

IR (ATR, ṽ) = 3478 (w), 3441 (w), 3429 (w), 3421 (w), 3393 (w), 3381 (w), 3371 (w), 3364 (w), 3350 (w), 3342 (w), 3332 (w), 3323 (w), 3308 (w), 3291 (w), 3286 (w), 3228 (w), 3218 (w), 3024 (w), 2924 (m), 2893 (w), 2851 (w), 1592 (w), 1492 (w), 1452 (m), 1438 (w), 1411 (w), 1366 (w), 1344 (w), 1320 (w), 1293 (w), 1261 (w), 1234 (w), 1184 (w), 1118 (w), 1061 (vs), 1034 (s), 956 (s), 933 (s), 898 (s), 849 (m), 795 (m), 759 (s), 730 (s), 715 (s), 700 (vs), 649 (m), 603 (m), 585 (m), 545 (s), 510 (vs), 475 (m), 459 (m), 442 (m), 429 (m), 412 (m), 387 (m), 375 (m) cm^{-1}.

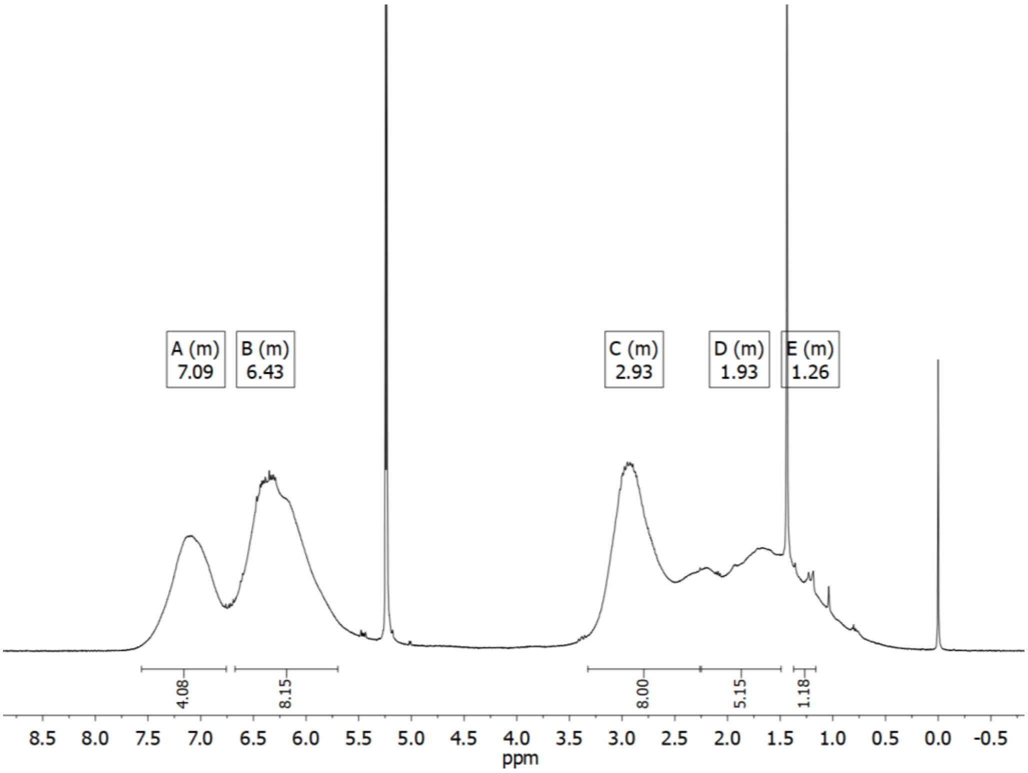

Figure S7. ^{1}H NMR of **9** synthesized using a 1:1 ratio.

Additional information on the chemical synthesis is available via the Chemotion repository: https://doi.org/10.14272/reaction/SA-FUHFF-UHFFFADPSC-IXIVTXOHXV-UHFFFADPSC-NUHFF-NUHFF-NUHFF-ZZZ.1 (accessed on 03.04.2025).

Additional information on the analysis of the target compound is available via the Chemotion repository: https://doi.org/10.14272/IXIVTXOHXVVPAC-UHFFFAOYSA-N.2 (accessed on 03.04.2025).

PCP:styrene 1:10:

^{1}H NMR (400 MHz, Dichloromethane-d_2 [5.32 ppm], ppm) δ = 7.21–6.92 (m, 32H, CH_{Ar}), 6.73–6.35 (m, 19H, CH_{Ar}), 6.33–5.98 (m, 4H, CH_{Ar}), 3.25–2.55 (m, 8H, ethylene bridge CH_2), 2.17–1.56 (m, 18H, alkyl backbone), 1.49–1.28 (m, 12H, alkyl backbone). Solvents: 7.24 + 7.15 + 2.34 ppm (toluene), 1.56 ppm (water).

GPC: M_n: 2.561*E^{+3} g/mol; M_w: 1.394*E^{+4} g/mol; D: 5.443*E^{+0};

IR (ATR, ṽ) = 3391 (vw), 3376 (vw), 3353 (vw), 3306 (vw), 3084 (vw), 3058 (vw), 3026 (w), 2922 (w), 2851 (w), 1601 (w), 1582 (vw), 1492 (w), 1452 (m), 1412 (vw), 1367 (w), 1346 (w), 1317 (vw), 1183 (w), 1156 (vw), 1115 (w), 1065 (m), 1030 (w), 982 (w), 962 (w), 935 (w), 904 (w), 863 (w), 844 (w), 796 (w), 756 (m), 697 (vs), 625 (w), 608 (w), 594 (w), 540 (m), 511

(w), 472 (w), 459 (w), 449 (w), 432 (w), 425 (w), 414 (w), 408 (w), 395 (w), 387 (w), 378 (w) cm^{-1}.

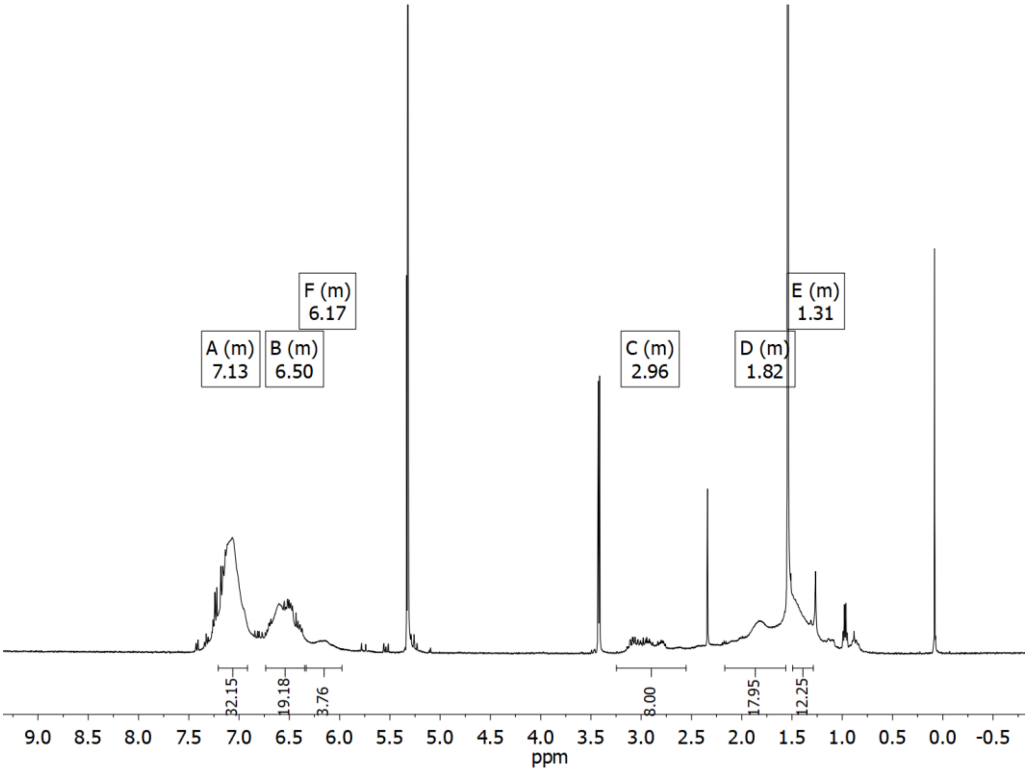

Figure S8. ^{1}H NMR of **9** synthesized using a 1:10 ratio.

Additional information on the chemical synthesis is available via the Chemotion repository: https://doi.org/10.14272/reaction/SA-FUHFF-UHFFFADPSC-IXIVTXOHXV-UHFFFADPSC-NUHFF-NUHFF-NUHFF-ZZZ.2 (accessed on 03.04.2025).

Additional information on the analysis of the target compound is available via the Chemotion repository: https://doi.org/10.14272/IXIVTXOHXVVPAC-UHFFFAOYSA-N.3 (accessed on 03.04.2025).

PCP:styrene 1:25:

^{1}H NMR (400 MHz, Dichloromethane-d_2 [5.32 ppm], ppm) δ = 7.29–6.87 (m, 94H, CH_{Ar}), 6.81–6.14 (m, 46H, CH_{Ar}), 3.32–2.57 (m, 8H, ethylene bridge CH_2), 2.24–1.52 (m, 46H, alkyl backbone), 1.50–0.83 (m, 31H, alkyl backbone). Solvents: 7.24 + 7.15 + 2.34 ppm (toluene), 1.56 ppm (water).

GPC: M_n: 4.682*E^{+3} g/mol; M_w: 1.064*E^{+4} g/mol; D: 2.272*E^{+0};

IR (ATR, ṽ) = 3429 (vw), 3364 (vw), 3303 (vw), 3058 (vw), 3024 (w), 2999 (vw), 2922 (w), 2850 (w), 1601 (w), 1492 (w), 1452 (w), 1412 (vw), 1367 (w), 1344 (vw), 1330 (vw), 1320 (vw), 1293 (vw), 1183 (w), 1154 (vw), 1118 (vw), 1065 (w), 1030 (w), 1001 (w), 963 (w), 935 (w), 922 (w), 908 (w), 844 (w), 756 (m), 697 (vs), 622 (w), 594 (w), 538 (m), 513 (w), 466 (w), 456 (w), 449 (w), 419 (w), 412 (w), 405 (w), 391 (vw), 384 (vw) cm^{-1}.

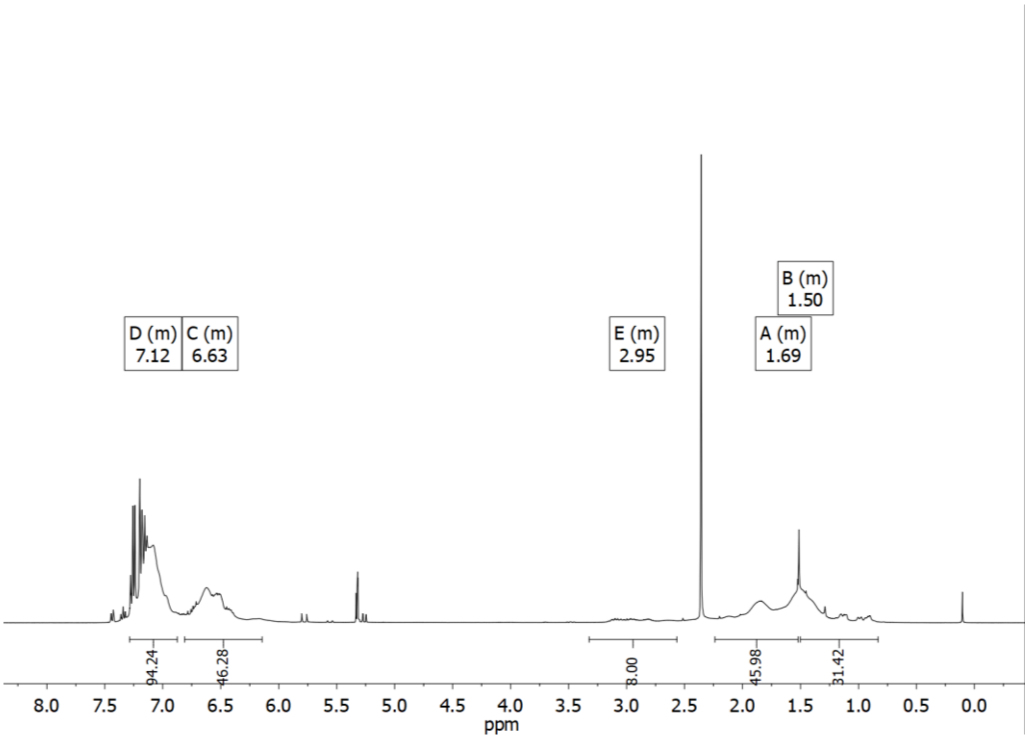

Figure S9. ^{1}H NMR of **9** synthesized using a 1:25 ratio.

Additional information on the chemical synthesis is available via the Chemotion repository: https://doi.org/10.14272/reaction/SA-FUHFF-UHFFFADPSC-IXIVTXOHXV-UHFFFADPSC-NUHFF-NUHFF-NUHFF-ZZZ.3 (accessed on 03.04.2025).

Additional information on the analysis of the target compound is available via the Chemotion repository: https://doi.org/10.14272/IXIVTXOHXVVPAC-UHFFFAOYSA-N.4 (accessed on 03.04.2025).

PCP:styrene 1:50:

^{1}H NMR (400 MHz, Dichloromethane-d_2 [5.32 ppm], ppm) δ = 7.30–6.92 (m, 147H, CH_{Ar}), 6.76–6.07 (m, 85H, CH_{Ar}), 3.24–2.54 (m, 8H, ethylene bridge CH_2), 2.20–1.55 (m, 72H, alkyl backbone), 1.48–0.85 (m, 52H, alkyl backbone). Solvents: 7.24 + 7.15 + 2.34 ppm (toluene), 1.56 ppm (water).

GPC: M_n: 6.015*E^{+3} g/mol; M_w: 1.369*E^{+4} g/mol; D: 2.276*E^{+0};

IR (ATR, ṽ) = 3427 (vw), 3350 (vw), 3301 (vw), 3087 (vw), 3061 (vw), 3026 (w), 2922 (w), 2851 (w), 1601 (w), 1493 (w), 1452 (w), 1367 (w), 1346 (vw), 1183 (w), 1153 (vw), 1065 (w), 1030 (w), 1003 (w), 962 (w), 935 (w), 922 (w), 908 (w), 844 (w), 755 (m), 697 (vs), 626 (w), 619 (w), 608 (w), 594 (w), 538 (m), 514 (w), 494 (w), 477 (w), 469 (w), 463 (w), 453 (w), 446 (w), 432 (w), 419 (w), 408 (w), 399 (w), 387 (w) cm^{-1}.

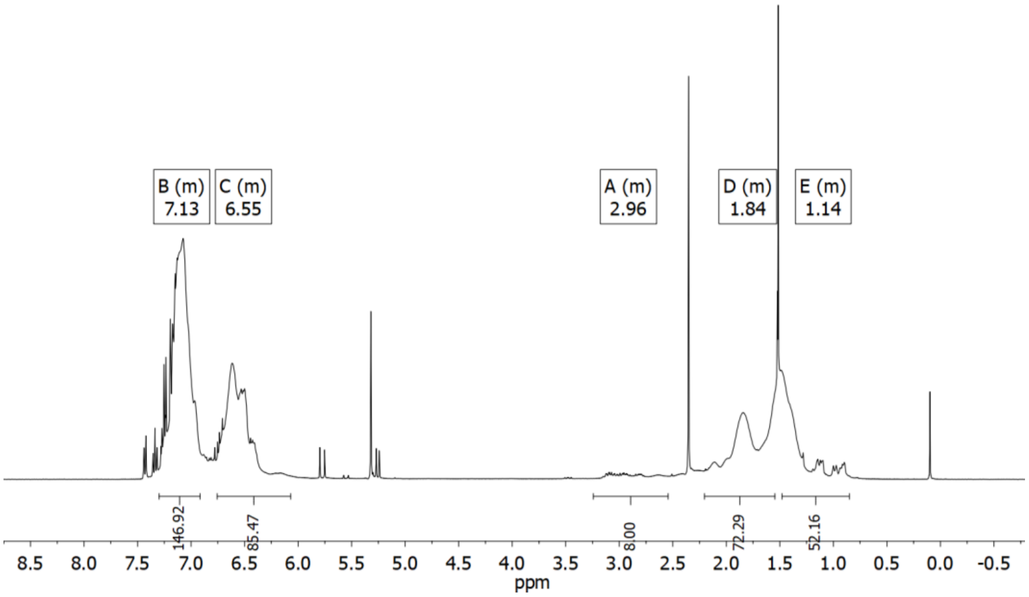

Figure S10. ^{1}H NMR of **9** synthesized using a 1:50 ratio.

Additional information on the chemical synthesis is available via the Chemotion repository: https://doi.org/10.14272/reaction/SA-FUHFF-UHFFFADPSC-IXIVTXOHXV-UHFFFADPSC-NUHFF-NUHFF-NUHFF-ZZZ (accessed on 03.04.2025).

Additional information on the analysis of the target compound is available via the Chemotion repository: https://doi.org/10.14272/IXIVTXOHXVVPAC-UHFFFAOYSA-N.1 (accessed on 03.04.2025).

2.3. General Procedure Copolymerization Vinyl-PCP + Vinyl-DMAC-TRZ (13)

In a Schlenk tube, 0.02 eq/monomer of the initiator AIBN was added to 10-(4-(4,6-diphenyl-1,3,5-triazin-2-yl)phenyl)-9,9-dimethyl-2-vinyl-9,10-dihydroacridine (1.00 equiv.) and 4-vinyl[2.2]paracyclophane (10.0 – 100 equiv.) in 0.10 mL of dry degassed toluene and the reaction heated to 80 °C to start the reaction. The reaction was cooled to 21 °C and terminated by precipitation by adding 5 mL of methanol after 24 h. The product was collected by centrifugation and redissolved in 0.250 mL of DCM. Afterward, it was precipitated by adding 5 mL of methanol, collected by centrifugation, and dried in a vacuum to yield poly(2-vinyl[2.2]paracyclophane-co-2-vinyl-DMAC-TRZ.

R_p-PCP:DMAC-TRZ 10:1:

^{1}H NMR (400 MHz, Dichloromethane-d_2 [5.32 ppm], ppm) δ = 9.17–8.47 (m, 6H, DMAC-TRZ-CH_{Ar}), 7.75–5.78 (m, 38H, CH_{Ar}), 3.86–2.40 (m, 30H, PCP-CH_2), 1.81–1.60 (m, 8H, alkyl backbone), 1.30–1.21 (m, 6H, alkyl backbone), 1.17–0.80 (m, 6H, alkyl backbone). Solvents: 1.56 ppm (water).

GPC: M_n: 1.166E^{+3} g/mol; M_w: 6.567E^{+3} g/mol; Đ: 5.633E^{+0};

IR (ATR, ṽ) = 3422 (w), 3405 (w), 3394 (w), 3388 (w), 3381 (w), 3361 (w), 3340 (w), 3332 (w), 2924 (m), 2853 (w), 1771 (w), 1721 (w), 1667 (w), 1588 (w), 1516 (s), 1476 (w), 1446 (m), 1411 (w), 1366 (s), 1323 (m), 1261 (m), 1241 (w), 1180 (s), 1057 (vs), 1033 (vs), 987 (vs), 921 (vs), 860 (s), 843 (s), 798 (vs), 772 (s), 745 (vs), 717 (s), 696 (s), 683 (s), 663 (s), 646 (s), 635 (s), 601 (s), 591 (s), 581 (s), 572 (s), 557 (s), 535 (s), 511 (s), 476 (s), 463 (s), 456 (s), 441 (s), 431 (m), 422 (m), 401 (s), 392 (s), 384 (s) cm^{-1}.

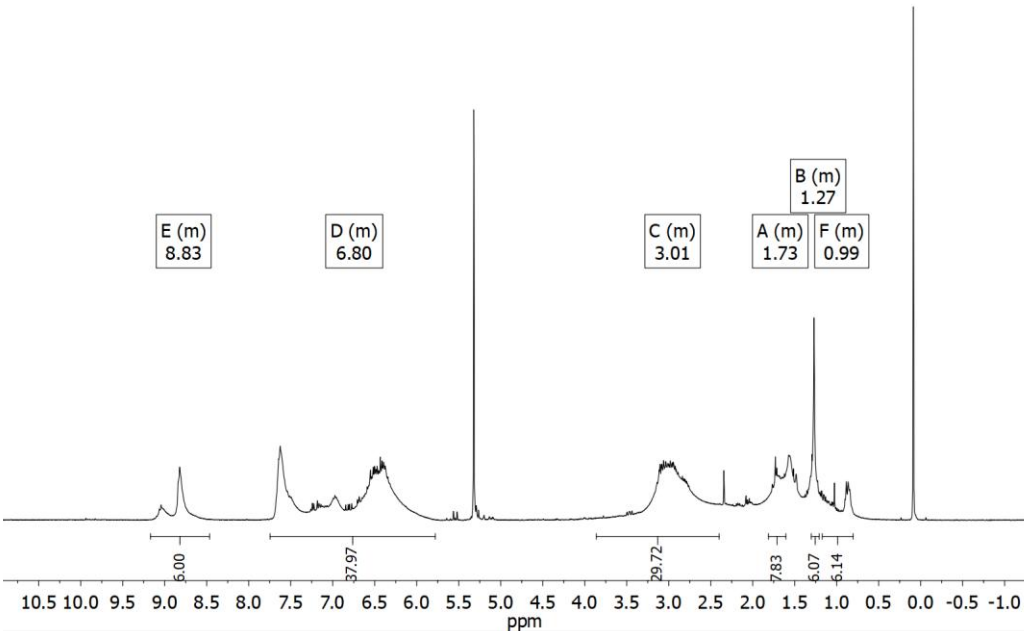

Figure S11. ^{1}H NMR of R_p-**13** synthesized using a 10:1 ratio.

Additional information on the chemical synthesis is available via the Chemotion repository: https://doi.org/10.14272/reaction/SA-FUHFF-UHFFFADPSC-JIWMTSMBOD-UHFFFADPSC-NUHFF-NUHFF-NUHFF-ZZZ.3 (accessed on 03.04.2025).

Additional information on the analysis of the target compound is available via the Chemotion repository: https://doi.org/10.14272/JIWMTSMBODORIT-UHFFFAOYSA-N.4 (accessed on 03.04.2025).

R_p -PCP:DMAC-TRZ 20:1:

^{1}H NMR (400 MHz, Dichloromethane-d_2 [5.32 ppm], ppm) δ = 9.21–8.67 (m, 6H, DMAC-TRZ-CH_{Ar}), 7.72–5.95 (m, 64H, CH_{Ar}), 3.63–2.39 (m, 53H, PCP-CH_2), 2.11–1.56 (m, 19H, alkyl backbone), 1.48–0.73 (m, 11H, alkyl backbone). Solvents: 1.56 ppm (water).

GPC: M_n: 2.428E^{+3} g/mol; M_w: 1.568E^{+4} g/mol; D: 6.458E^{+0};

IR (ATR, ṽ) = 3509 (vw), 3495 (w), 3397 (w), 3353 (w), 3337 (w), 3330 (w), 3320 (w), 3312 (w), 3281 (w), 3250 (w), 2955 (w), 2925 (w), 2893 (w), 2853 (w), 1721 (w), 1639 (w), 1588 (w), 1554 (vw), 1517 (m), 1476 (w), 1445 (m), 1411 (m), 1366 (s), 1344 (m), 1322 (m), 1261 (m), 1188 (m), 1118 (m), 1058 (vs), 1034 (vs), 960 (vs), 925 (vs), 899 (s), 843 (s), 798 (vs), 772 (s), 745 (s), 730 (s), 717 (s), 696 (s), 684 (s), 663 (s), 646 (s), 636 (s), 603 (s), 557 (s), 534 (s), 511 (vs), 484 (s), 452 (s), 438 (s), 426 (s), 411 (s), 392 (s), 384 (s), 377 (s) cm^{-1}.

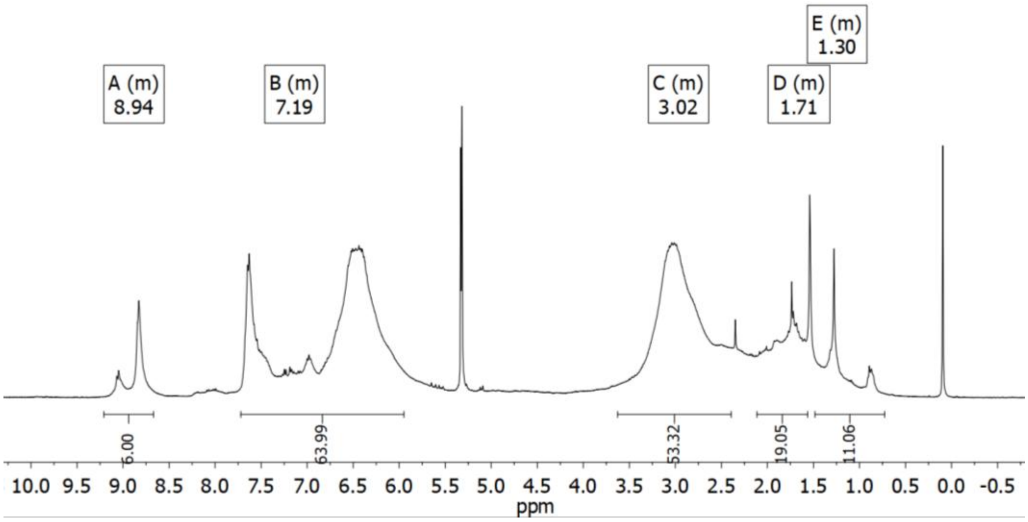

Figure S12. ^{1}H NMR of R$_P$-**13** synthesized using a 20:1 ratio.

Additional information on the chemical synthesis is available via the Chemotion repository: https://doi.org/10.14272/reaction/SA-FUHFF-UHFFFADPSC-JIWMTSMBOD-UHFFFADPSC-NUHFF-NUHFF-NUHFF-ZZZ (accessed on 03.04.2025).

Additional information on the analysis of the target compound is available via the Chemotion repository: https://doi.org/10.14272/JIWMTSMBODORIT-UHFFFAOYSA-N.1 (accessed on 03.04.2025).

R$_p$ -PCP:DMAC-TRZ 50:1:

^{1}H NMR (400 MHz, Dichloromethane-d$_2$ [5.32 ppm], ppm) δ = 9.07–8.79 (m, 6H, DMAC-TRZ-CH$_{Ar}$), 7.95–5.73 (m, 92H, CH$_{Ar}$), 3.84–2.58 (m, 84H, PCP-CH$_2$), 2.18–1.64 (m, 23H, alkyl backbone), 1.39–0.69 (m, 115H, alkyl backbone + unknown impurity). Solvents: 1.56 ppm (water).

GPC: M$_n$: 8.888E^{+2} g/mol; M$_w$: 3.977E^{+3} g/mol; D: 4.474E^{+0};

IR (ATR, ṽ) = 3401 (w), 3384 (w), 3377 (w), 3370 (w), 3359 (w), 3346 (w), 3329 (w), 3284 (w), 3269 (w), 3257 (w), 2925 (m), 2893 (w), 2853 (w), 1771 (w), 1721 (w), 1664 (w), 1656 (w), 1647 (w), 1642 (w), 1619 (w), 1589 (w), 1517 (w), 1482 (w), 1445 (m), 1411 (m), 1366 (m), 1343 (m), 1322 (m), 1289 (w), 1261 (m), 1239 (w), 1181 (s), 1057 (vs), 1034 (vs), 986 (vs), 958 (vs), 922 (vs), 899 (vs), 858 (s), 850 (s), 846 (s), 798 (vs), 773 (s), 745 (s), 730 (s), 717 (s), 697 (s), 684 (s), 646 (s), 637 (s), 603 (s), 596 (s), 589 (s), 584 (s), 574 (s), 558 (s), 538 (s), 530 (s), 511 (vs), 477 (s), 469 (s), 452 (s), 443 (s), 428 (m), 419 (m), 405 (s), 395 (m), 387 (s), 375 (m) cm^{-1}.

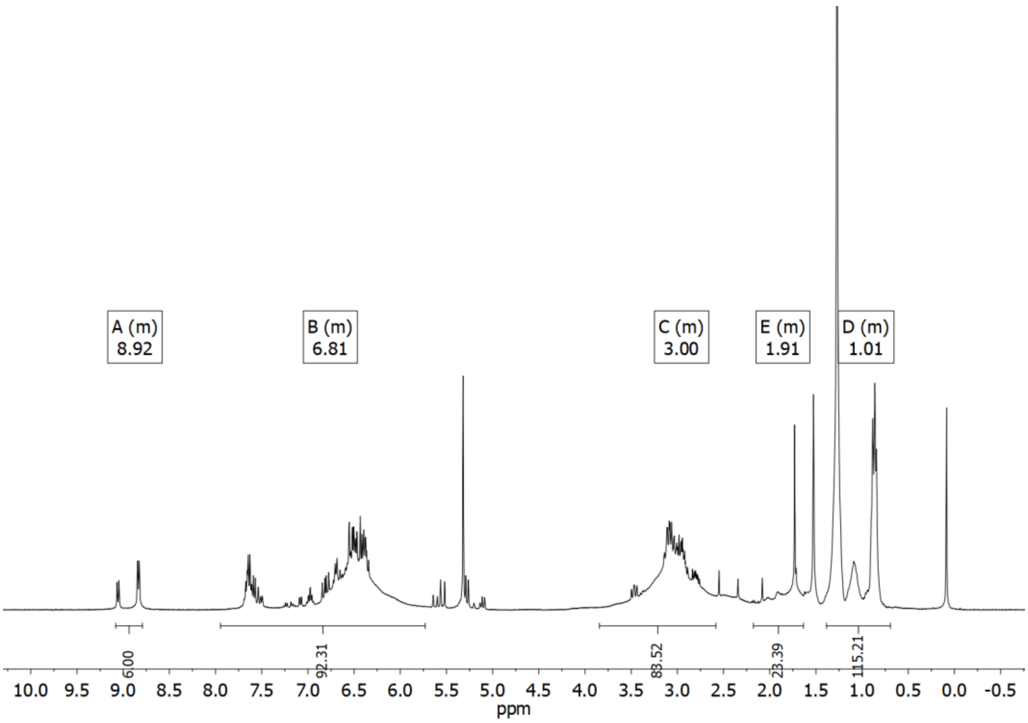

Figure S13. ^{1}H NMR of R_p-**13** synthesized using a 50:1 ratio.

Additional information on the chemical synthesis is available via the Chemotion repository: https://doi.org/10.14272/reaction/SA-FUHFF-UHFFFADPSC-JIWMTSMBOD-UHFFFADPSC-NUHFF-NUHFF-NUHFF-ZZZ.2 (accessed on 03.04.2025).

Additional information on the analysis of the target compound is available via the Chemotion repository: https://doi.org/10.14272/JIWMTSMBODORIT-UHFFFAOYSA-N.3 (accessed on 03.04.2025).

R_p -PCP:DMAC-TRZ 100:1:

^{1}H NMR (400 MHz, Dichloromethane-d$_2$ [5.32 ppm], ppm) δ = 9.11–8.71 (m, 6H, DMAC-TRZ-CH$_{Ar}$), 7.79–5.76 (m, 148H, CH$_{Ar}$), 3.58–2.39 (m, 145H, PCP-CH$_2$), 2.14–1.66 (m, 21H, alkyl backbone), 1.44–0.66 (m, 55H, alkyl backbone). Solvents: 1.56 ppm (water).

GPC: M_n: 5.073E^{+2} g/mol; M_w: 1.955E^{+3} g/mol; D: 3.854E^{+0};

IR (ATR, ṽ) = 3442 (w), 3431 (w), 3410 (w), 3394 (w), 3377 (w), 3359 (w), 3347 (w), 3332 (w), 3299 (w), 3264 (w), 3208 (w), 2956 (m), 2922 (m), 2851 (w), 1744 (vw), 1720 (w), 1710 (w), 1656 (w), 1639 (w), 1626 (w), 1589 (w), 1553 (w), 1541 (w), 1517 (m), 1446 (m), 1411 (m), 1366 (m), 1344 (m), 1322 (m), 1286 (w), 1259 (s), 1188 (m), 1061 (vs), 1034 (vs), 963 (vs), 925 (vs), 899 (s), 860 (s), 843 (s), 796 (vs), 745 (s), 731 (s), 717 (s), 697 (s), 684 (s), 662 (s), 646 (m), 606 (s), 586 (m), 561 (s), 554 (s), 511 (s), 482 (m), 448 (m), 436 (m), 431 (m), 395 (s) cm^{-1}.

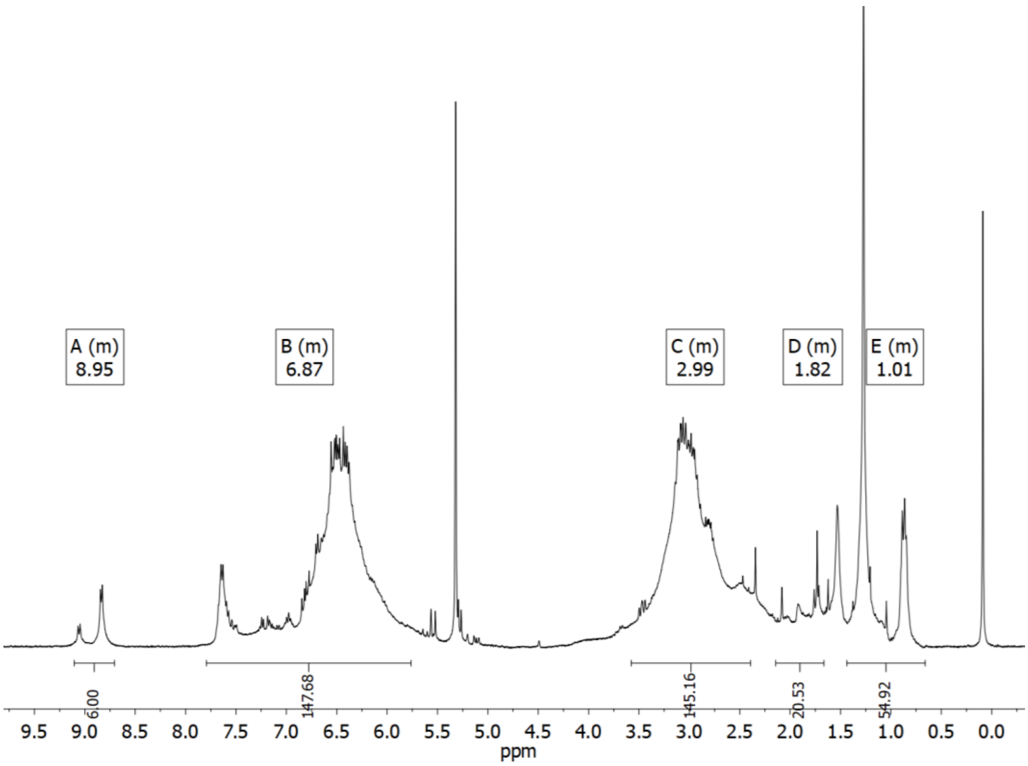

Figure S14. ^{1}H NMR of R_p-**13** synthesized using a 100:1 ratio.

Additional information on the chemical synthesis is available via the Chemotion repository: https://doi.org/10.14272/reaction/SA-FUHFF-UHFFFADPSC-JIWMTSMBOD-UHFFFADPSC-NUHFF-NUHFF-NUHFF-ZZZ.1 (accessed on 03.04.2025).

Additional information on the analysis of the target compound is available via the Chemotion repository: https://doi.org/10.14272/JIWMTSMBODORIT-UHFFFAOYSA-N.2 (accessed on 03.04.2025).

S_p -PCP:DMAC-TRZ 20:1:

^{1}H NMR (400 MHz, Dichloromethane-d_2 [5.32 ppm], ppm) δ = 9.15–8.65 (m, 6H, DMAC-TRZ-CH_{Ar}), 7.76–5.79 (m, 76H, CH_{Ar}), 3.56–2.56 (m, 63H, PCP-CH_2), 2.04–1.65 (m, 15H, alkyl backbone), 1.37–0.79 (m, 32H, alkyl backbone). Solvents: 1.56 ppm (water).

GPC: M_n: 7.335E^{+2} g/mol; M_w: 2.190E^{+3} g/mol; D: 2.986E^{+0}.

IR (ATR, ṽ) = 3031 (w), 3004 (w), 2921 (vs), 2894 (s), 2850 (s), 2772 (w), 1720 (w), 1684 (w), 1679 (w), 1588 (m), 1514 (vs), 1480 (m), 1446 (s), 1411 (m), 1366 (vs), 1322 (m), 1268 (m), 1230 (w), 1201 (w), 1176 (m), 1157 (m), 1096 (w), 1085 (w), 1067 (w), 1050 (w), 1024 (m), 1017 (m), 1000 (w), 936 (w), 897 (s), 863 (m), 843 (m), 795 (vs), 771 (s), 744 (vs), 730 (s), 715 (vs), 696 (s), 684 (m), 663 (w), 646 (s), 636 (m), 606 (m), 586 (w), 509 (vs) cm^{-1}.

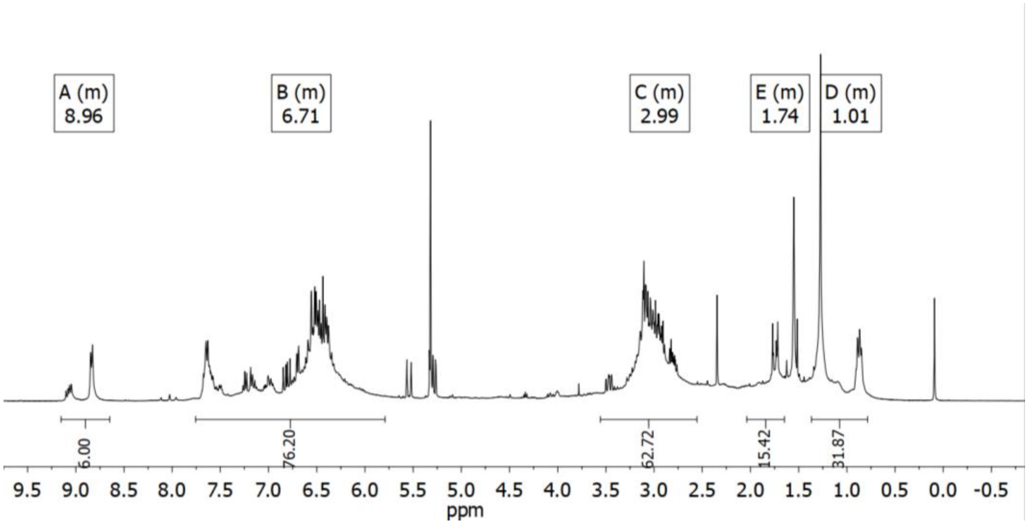

Figure S15. ^{1}H NMR of S_p-**13** synthesized using a 20:1 ratio.

Additional information on the chemical synthesis is available via the Chemotion repository: https://doi.org/10.14272/reaction/SA-FUHFF-UHFFFADPSC-JIWMTSMBOD-UHFFFADPSC-NUHFF-NUHFF-NUHFF-ZZZ.4 (accessed on 03.04.2025).

Additional information on the analysis of the target compound is available via the Chemotion repository: https://doi.org/10.14272/JIWMTSMBODORIT-UHFFFAOYSA-N.5 (accessed on 03.04.2025).

S_p -PCP:DMAC-TRZ 50:1:

^{1}H NMR (400 MHz, Dichloromethane-d$_2$ [5.32 ppm], ppm) δ = 9.24–8.66 (m, 6H, DMAC-TRZ-CH$_{Ar}$), 7.87–5.77 (m, 162H, CH$_{Ar}$), 3.34–2.57 (m, 142H, PCP-CH$_2$), 1.87–1.64 (m, 22H, alkyl backbone), 1.43–0.70 (m, 148H, alkyl backbone). Solvents: 1.56 ppm (water).

GPC: M_n: 7.566E^{+2} g/mol; M_w: 2.272E^{+3} g/mol; D: 3.003E^{+0};

IR (ATR, ṽ) = 3469 (w), 3429 (w), 3401 (w), 3384 (w), 3377 (w), 3370 (w), 3359 (w), 3346 (w), 3329 (w), 3284 (w), 3269 (w), 3257 (w), 2955 (m), 2925 (m), 2893 (w), 2853 (w), 1771 (w), 1721 (w), 1664 (w), 1656 (w), 1647 (w), 1642 (w), 1619 (w), 1589 (w), 1517 (w), 1482 (w), 1445 (m), 1411 (m), 1366 (m), 1343 (m), 1322 (m), 1289 (w), 1261 (m), 1239 (w), 1181 (s), 1057 (vs), 1034 (vs), 986 (vs), 958 (vs), 922 (vs), 899 (vs), 858 (s), 850 (s), 846 (s), 798 (vs), 773 (s), 745 (s), 730 (s), 717 (s), 697 (s), 684 (s), 646 (s), 637 (s), 603 (s), 596 (s), 589 (s), 584 (s), 574 (s), 558 (s), 538 (s), 530 (s), 511 (vs), 477 (s), 469 (s), 452 (s), 443 (s), 428 (m), 419 (m), 405 (s), 395 (m), 387 (s), 375 (m) cm^{-1}.

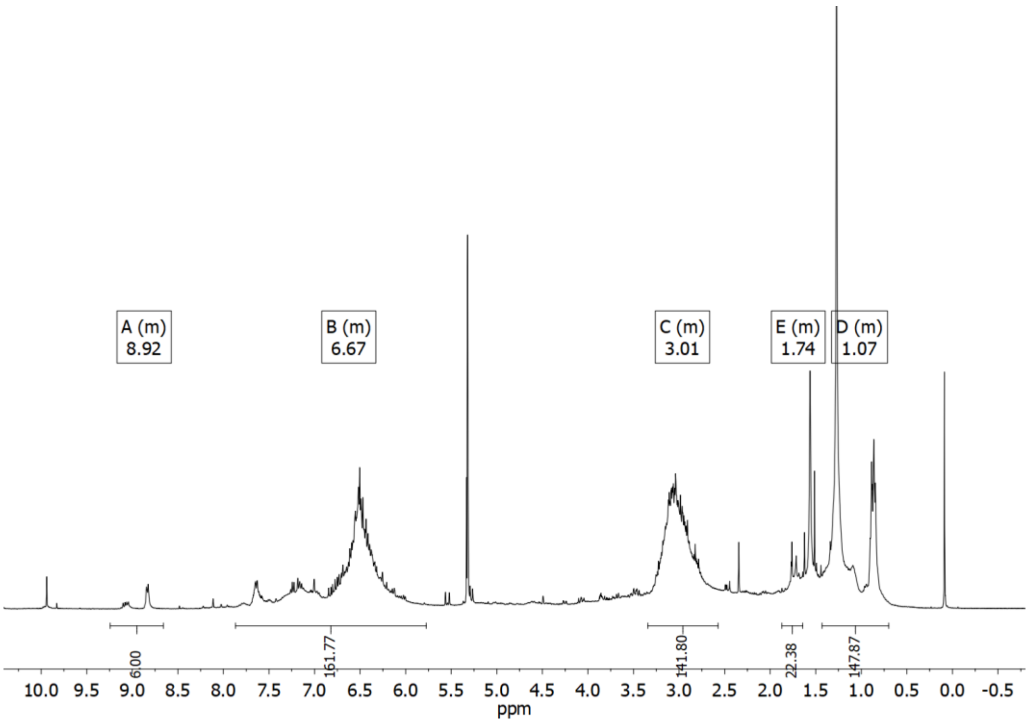

Figure S16. ^{1}H NMR of S$_P$-**13** synthesized using a 50:1 ratio.

Additional information on the chemical synthesis is available via the Chemotion repository: https://doi.org/10.14272/reaction/SA-FUHFF-UHFFFADPSC-JIWMTSMBOD-UHFFFADPSC-NUHFF-NUHFF-NUHFF-ZZZ.5 (accessed on 03.04.2025).

Additional information on the analysis of the target compound is available via the Chemotion repository: https://doi.org/10.14272/JIWMTSMBODORIT-UHFFFAOYSA-N.6 (accessed on 03.04.2025).

2.4. Procedure Polymerization Poly(Vinyl-DMAC-TRZ) (14)

In a Schlenck tube, AIBN (1.13 mg, 6.91 µmol, 0.0500 equiv.) was added to 2-vinyl-DMAC-TRZ (75.0 mg, 138 µmol, 1.00 equiv.) in 0.20 mL of dry degassed toluene and the reaction heated to 80 °C to start the reaction. After 24 h, the reaction was cooled to 21 °C and terminated by injecting 5 mL of methanol. The product was collected by centrifugation and redissolved in 0.25 mL of DCM. Afterwards, it was precipitated again by adding 5 mL of methanol to yield poly-vinyl-DMAC-TRZ (25.1 mg, 33% yield).

^{1}H NMR (400 MHz, Dichloromethane-d$_2$ [5.32 ppm], ppm) δ = 9.10–8.69 (m, 6H, CH$_{Ar}$), 8.07–7.87 (m, 1H, CH$_{Ar}$), 7.69–7.30 (m, 10H, CH$_{Ar}$), 7.10–6.86 (m, 2H, CH$_{Ar}$), 6.47–6.25 (m, 2H, CH$_{Ar}$), 1.78–0.84 (m, 9H, DMAC-CH$_3$ + alkyl backbone). Solvent: 3.42 + 1.09 ppm (methanol).

GPC: M$_n$: 1.456E^{+3} g/mol; M$_w$: 2.711E^{+3} g/mol; D: 1.862E^{+0}.

IR (ATR, ṽ) = 3064 (w), 3057 (w), 3040 (w), 3031 (w), 2961 (w), 2917 (w), 2854 (w), 1718 (w), 1599 (w), 1587 (w), 1510 (vs), 1475 (s), 1445 (vs), 1408 (w), 1363 (vs), 1320 (s), 1264 (vs), 1193 (w), 1173 (m), 1146 (w), 1106 (m), 1096 (m), 1068 (m), 1050 (w), 1024 (m), 1017 (m), 1001 (w), 989 (w), 973 (w), 929 (w), 901 (w), 887 (w), 874 (w), 841 (m), 830 (w), 813 (m), 769 (s), 742 (vs), 710 (s), 691 (vs), 683 (vs), 663 (m), 646 (s), 636 (m), 618 (w), 603 (w), 571 (w), 557 (w), 517 (m), 489 (w), 466 (w), 450 (w), 439 (w), 428 (w), 416 (w), 409 (w), 404 (w), 390 (w), 375 (w) cm^{-1}.

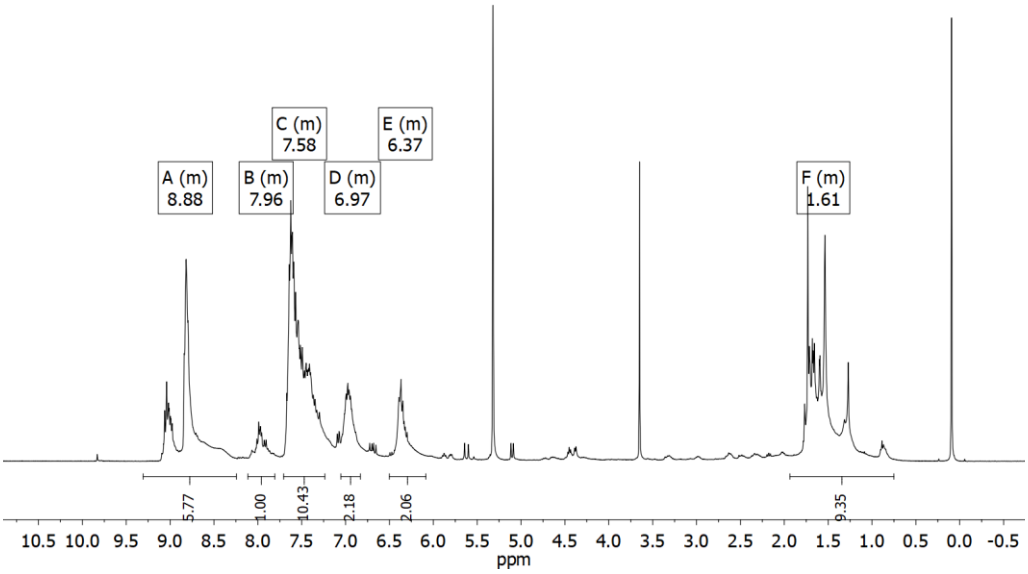

Figure S17. ^{1}H NMR of 14.

Additional information on the chemical synthesis is available via the Chemotion repository: https://doi.org/10.14272/reaction/SA-FUHFF-UHFFFADPSC-UHFFFADPSC-UHFFFADPSC-NUHFF-NUHFF-NUHFF-ZAZ.9 (accessed on 03.04.2025).

Additional information on the analysis of the target compound is available via the Chemotion repository: https://doi.org/10.14272/10.14272/DUMMY.8 (accessed on 03.04.2025).

References

1. Zippel, C.; Hassan, Z.; Parsa, A.Q.; Hohmann, J.; Bräse, S. Multigram-Scale Kinetic Resolution of 4-Acetyl [2.2] Paracyclophane via Ru-Catalyzed Enantioselective Hydrogenation: Accessing [2.2] Paracyclophanes with Planar and Central Chirality. *Adv. Synth. Catal.* **2021**, *363*, 2861–2865.

Disclaimer/Publisher's Note: The statements, opinions and data contained in all publications are solely those of the individual author(s) and contributor(s) and not of MDPI and/or the editor(s). MDPI and/or the editor(s) disclaim responsibility for any injury to people or property resulting from any ideas, methods, instructions or products referred to in the content.
